# TbSAP is a novel chromatin protein repressing metacyclic variant surface glycoprotein expression sites in bloodstream form *Trypanosoma brucei*

**DOI:** 10.1093/nar/gkab109

**Published:** 2021-02-28

**Authors:** Carys Davies, Cher-Pheng Ooi, Georgios Sioutas, Belinda S Hall, Haneesh Sidhu, Falk Butter, Sam Alsford, Bill Wickstead, Gloria Rudenko

**Affiliations:** Sir Alexander Fleming Building, Department of Life Sciences, Imperial College London, South Kensington, London SW7 2AZ, UK; Sir Alexander Fleming Building, Department of Life Sciences, Imperial College London, South Kensington, London SW7 2AZ, UK; Sir Alexander Fleming Building, Department of Life Sciences, Imperial College London, South Kensington, London SW7 2AZ, UK; Sir Alexander Fleming Building, Department of Life Sciences, Imperial College London, South Kensington, London SW7 2AZ, UK; Sir Alexander Fleming Building, Department of Life Sciences, Imperial College London, South Kensington, London SW7 2AZ, UK; Institute of Molecular Biology, Ackermannweg 4, 55128 Mainz, Germany; London School of Hygiene and Tropical Medicine, Keppel Street, London WC1E 7HT, UK; School of Life Sciences, Queens Medical Centre, University of Nottingham, Nottingham NG7 2UH, UK; Sir Alexander Fleming Building, Department of Life Sciences, Imperial College London, South Kensington, London SW7 2AZ, UK

## Abstract

The African trypanosome *Trypanosoma brucei* is a unicellular eukaryote, which relies on a protective variant surface glycoprotein (VSG) coat for survival in the mammalian host. A single trypanosome has >2000 *VSG* genes and pseudogenes of which only one is expressed from one of ∼15 telomeric bloodstream form expression sites (BESs). Infectious metacyclic trypanosomes present within the tsetse fly vector also express *VSG* from a separate set of telomeric metacyclic ESs (MESs). All MESs are silenced in bloodstream form *T. brucei*. As very little is known about how this is mediated, we performed a whole genome RNAi library screen to identify MES repressors. This allowed us to identify a novel SAP domain containing DNA binding protein which we called TbSAP. TbSAP is enriched at the nuclear periphery and binds both MESs and BESs. Knockdown of TbSAP in bloodstream form trypanosomes did not result in cells becoming more ‘metacyclic-like'. Instead, there was extensive global upregulation of transcripts including MES *VSG*s, *VSG*s within the silent *VSG* arrays as well as genes immediately downstream of BES promoters. TbSAP therefore appears to be a novel chromatin protein playing an important role in silencing the extensive *VSG* repertoire of bloodstream form *T. brucei*.

## INTRODUCTION

The African trypanosome *Trypanosoma brucei* (causative agent of African Sleeping sickness) is an early-branching unicellular eukaryote with highly unusual features to its molecular biology (1). The *T. brucei* genome consists primarily of extensive polycistronic transcription units containing functionally unrelated assortments of genes which are constitutively transcribed by RNA polymerase II (Pol II) (2). The *T. brucei* genome encodes relatively few transcription factors compared with other eukaryotes (3), which is consistent with the observed general lack of Pol II transcriptional regulation (4,5). Instead, regulation of mRNA levels during the trypanosome life-cycle or in response to stress, occurs primarily through post-transcriptional mechanisms including RNA stability mediated through sequence elements located in mRNA untranslated regions (6).

The striking exception to this rule is the transcriptional regulation of the vast number of *T. brucei* variant surface glycoprotein (VSG) genes. *T. brucei* is an extracellular parasite of the mammalian bloodstream and tissue spaces (7,8). Within the host, it is covered with a dense protective VSG coat comprised of ∼10^7^ molecules corresponding to ∼10% total protein (9). A single trypanosome has a vast repertoire of thousands of *VSG* genes and pseudogenes, but only one is transcribed at a time from one of 15 bloodstream form *VSG* expression sites (BES) (10,11). BESs are telomeric transcription units with promoters located 30–60 kb upstream of the telomeric *VSG*, and which include various expression site associated genes (ESAGs) (12). Switching the active *VSG* entails replacement with another *VSG* through DNA rearrangements including gene conversion, or a transcriptional switch to a different BES (13–16).

A highly unusual feature of BES transcription is that it is mediated by RNA polymerase I (Pol I) (17). This use of Pol I for transcription of protein coding genes is unprecedented for a eukaryote, as Pol I normally exclusively transcribes ribosomal DNA (rDNA) (18). Pol I transcription is characterised by extremely high rates of transcription initiation (19). This feature of Pol I appears to facilitate the ability of the trypanosome to produce vast amounts of *VSG* transcript (about 10% total mRNA) from a single copy *VSG* gene. Pol I transcription of rDNA in eukaryotes is concentrated within a nuclear body referred to as the nucleolus (20). This is also the case in *T. brucei*, but bloodstream form trypanosomes additionally contain an extra-nucleolar Pol I focus called the expression site body (ESB), where transcription of the active BES occurs (21). Here, a highly stringent mechanism of monoallelic expression operates, with restricted access of the ESB to a single BES playing a key role in this control (22).

However, inactive BESs are not the only ESs which are kept silent in bloodstream form trypanosomes. *T. brucei* is transmitted by tsetse flies, and the infectious metacyclic stage present in the fly salivary glands is covered with a VSG coat transcribed from one of several metacyclic expression sites (MESs). MESs differ in architecture to BESs, with MES promoters normally within 1–2 kb of the telomeric *VSG*, compared with the 30–60 kb found in BESs (10,12). In addition, MES promoters have divergent sequences compared with BES promoters (23). However, both ES promoter types are transcribed by Pol I, and are recognised by the same CITFA Pol I transcription factor (24).

For antigenic variation to work, it is vital that both BESs and MESs are kept silent in addition to the vast number of *VSG* genes within the subtelomeric *VSG* arrays which are not thought to be flanked by promoters. Little is known about how all MESs are kept silent in bloodstream form *T. brucei*. Promoter sequence alone is not sufficient to explain MES silencing, as MES promoters can function in bloodstream form *T. brucei* when present on episomes, but not when they are in their native telomeric MES location (23). We set out to identify factors playing a functional role in silencing telomeric MESs in bloodstream form *T. brucei*. To this end, we performed a whole genome RNAi library screen selecting for derepression of a single inactive MES marked with a drug resistance gene.

We did not identify known BES repressors, but discovered a novel SAP DNA binding protein (TbSAP), which plays an important role in MES repression. We found that TbSAP is enriched at the *T. brucei* nuclear periphery, and using ChIP, established that it binds MESs as well as immediately upstream of BES promoters. Knockdown of TbSAP resulted in extensive global upregulation of *T. brucei* transcripts arguing that it has a general repressive function. These derepressed transcripts included transcripts from MES *VSGs*, *VSG*s within the silent *VSG* arrays, as well as genes in the immediate region of BES promoters. TbSAP therefore appears to be a novel chromatin protein with a key functional role in repression of the extensive *VSG* repertoire of bloodstream form *T. brucei*.

## MATERIALS AND METHODS

### Trypanosome strain generation and culturing

Bloodstream form (BF) *Trypanosoma brucei* strain 427 expressing *VSG221* from BES1 (12) was used for all experiments, and cultured in modified HMI-9 medium supplemented with 15% foetal calf serum. All cell lines used in this study are detailed in Supplementary Table S1, and are all based on the ‘single marker’ (SM) cell line (25) which is referred to here as SM221. The SM221pur cell line has a puromycin resistance gene inserted immediately behind the BES1 promoter allowing selection for maintenance of VSG221 expression (26). All genetically modified cell lines were validated using linking PCR.

To mark an individual MES in BF *T. brucei*, constructs were inserted downstream of the endogenous promoter of MES *VSG653* (10). The *T. brucei* SM221 MES-pur cell line was generated by transfecting the pMES653PurBla plasmid into SM221 cells resulting in a puromycin resistance gene inserted downstream of the endogenous MES *VSG653* promoter. The *T. brucei* SM221 MES-GFP cell line was generated through the transfection of the pMES653eGFPBla plasmid into *T. brucei* SM221 cells resulting in an eGFP gene inserted downstream of the endogenous MES *VSG653* promoter. In both cell lines, construct integration was selected for using a blasticidin resistance driven by an ectopic rDNA promoter. Both plasmids were generated by Gibson assembly using the primers listed in Supplementary Table S2.

*T. brucei* SM221pur Cas9 cells constitutively expressing Cas9 endonuclease were generated by transfecting the pSMOx2 Cas9 plasmid (derived from a plasmid kindly provided by Jack Sunter, Oxford Brookes University, UK). This integrates a codon-optimised Cas9 gene upstream of the first β-tubulin gene in the tubulin array, which is expressed by endogenous Pol II transcription. Cas9 mediated gene tagging and gene knock-out were carried out as described in (27). pPOT plasmids for this purpose were kindly provided by Sam Dean, (University of Warwick, UK) and primers were designed using the LeishGEedit website (www.leishgedit.net). All plasmids and primers used for Cas9-mediated gene tagging and knock-out are listed in Supplementary Table S2.

Stem–loop RNAi constructs were generated by cloning a PEX11 stuffer fragment flanked upstream by HindIII/XbaI and downstream by BamHI/ AscI into pDexv4-mSt-KIN17 (from the Bill Wickstead lab). A PEX11 stuffer was cloned downstream of a tetracycline inducible T7 promoter. Complementary gene sequences were cloned flanking this PEX11 stuffer, and the construct was inserted into the *VSGG4* locus (Tb427VSG-31, Tb427_00805900) on a *T. brucei* mini-chromosome (28) after digestion with NotI and transfection into SM221pur cells.

All RNAi cell lines were generated from SM221pur MES-GFP cells, with the exception of the α-tubulin RNAi cell line which was generated using *T. brucei* BNII V02+ pSMOx2. This cell line was generated using the BNII V02+ cell line (29) transfected with a derivative of the pSMOx plasmid containing codon optimised tetracycline repressor and T7 RNA polymerase (30) with alterations allowing construct integration upstream of the first β-tubulin gene of the tubulin array (kind gift of Mark Carrington, University of Cambridge, UK). PFR2 (Tb927.8.4970) was targeted for RNAi using the first 400 bp of its CDS, TbSAP was targeted for RNAi from position 130 to 634 of its CDS and α-tubulin was targeted for RNAi from position 629 to 1340 of its CDS.

### RNAi library generation and selection for repressors of MES transcription

The *T. brucei* SM221MES-pur cell line was transfected with pRP^Sce*^. This construct was generated following transfer of the Sce* cassette, containing the I-SceI meganuclease and its recognition site, from pRPa^Sce*^(31) to the pRP construct via HindIII/ Bsp120I cloning (32). In contrast to pRPa^Sce*^, which targets an ectopic rDNA spacer landing pad locus in 2T1 *T. brucei* (32), pRP^Sce*^ is able to integrate at any of the conserved rDNA spacer sequences in the SM221MES-pur *T. brucei* cell line. Transformants were selected with hygromycin.

pRPa^Sce*^ Hyg transfected cells were pooled for a total number of ∼3 × 10^7^ cells, and incubated for 24 h without hygromycin before transfection of the *T. brucei* whole genome RNAi plasmid library from (33). Transfection of the plasmid RNAi library was performed after 3 h of tetracycline induction to induce I-SceI expression and target site cleavage, enabling high efficiency integration of the RNAi library at the I-SceI cleaved rDNA spacer sequence (31). Phleomycin was added 5 h after transfection to select for RNAi library integration. *T. brucei* RNAi library cells were counted daily and expanded continuously until total cell number exceeded 1 × 10^8^. Screens for repressors of MES expression were initiated directly from expanded RNAi library cells using 2 × 10^7^ cells per screen. The RNAi library was induced with tetracycline for 24 h to ensure robust RNAi target depletion prior to addition of puromycin selection (50, 60 or 70 ng/ml). Genomic DNA for high throughput RIT-seq analysis was harvested (Blood and Tissue Kit, Qiagen) from the 50 ng or 60 ng/ml puromycin screens following ten days under selection, and from the 70 ng/ml screen after 23 days under selection. The genomic DNA from library cells grown in the absence of tetracycline or puromycin was harvested after either three or ten days of growth.

RIT-seq (RNA interference target sequencing) was carried out as previously described (31). Briefly, the remaining RNAi target fragments in each selected RNAi library were PCR amplified using LIB2f and LIB2r primers as described in (34). The amplified products were sequenced on an Illumina MiSeq platform (BGI Genomics). Sequence reads containing the RNAi construct-specific 14-base barcode were identified using a custom script (34). Mapping was carried out using Bowtie2 (35) set to ‘very sensitive local’ alignment, and output SAM files were processed using SAMtools (36). The resultant BAM files were viewed against the reference genome in the Artemis genome browser (37). RNAi target fragment read numbers were converted to RPKM (reads per kilobase per million reads mapped) to account for interlibrary read depth variations when comparing sequencing outputs from independently selected RNAi libraries.

### Immunofluorescence microscopy

In order to visualise target proteins using antibodies, cells were washed twice in cold PSG buffer and fixed in 2% formaldehyde for 15 minutes at room temperature. Fixed cells were washed twice in PBS and settled on ColorFrost Plus microscopy slides (Shandon) for 30 min in a humidity chamber. Cells were then permeabilised using 0.1% NP-40 and then subsequently incubated with the following primary antibodies: A rabbit polyclonal anti-GFP antibody (Abcam, Ab290) and anti-Ty epitope mouse monoclonal BB2 (kind gift of the Keith Gull lab). The secondary antibodies used were: goat anti-rabbit IgG (H+L) conjugated with Dylight 488, or goat anti-mouse IgG (H+L) conjugated to Dylight 594 (both from ThermoFisher Scientific). Cells were then mounted in Vectashield with DAPI (Vector Laboratories) and imaged on a Zeiss M1 Imager microscope with an AxioCam MRm camera. Post-acquisition analyses were carried out using ImageJ with the BAR plug-in for quantitation of signal intensity (NIH, Bethesda, MD, USA). Sample preparation for visualisation of telomeres using DNA FISH was carried out as detailed above, although instead of antibodies, the DAKO Telomere PNA FISH kit/FITC (Agilent) was used according to the manufacturer's instructions.

### Chromosome immunoprecipitation (ChIP) experiments

ChIP analysis of TbSAP and histone H3 was carried out as described in (38) and (22) with minor modifications. *T. brucei* TbSAP-eGFP/TbSAP KO or untagged SM221pur cells were cross-linked with 1% formaldehyde for 1 h at room temperature before the reaction was quenched by glycine addition to a final concentration of 125 mM. Cross-linked cells were sonicated (BioRuptor, Diagenode) to obtain an average DNA fragment size of 200 bp. Immunoprecipitation was carried out by incubation at 4°C for 18 h with anti-GFP (Ab290, Abcam) or anti-histone H3 (Ab1791, Abcam) antibodies bound to Protein G magnetic Dynabeads (ThermoFisher). Dynabeads without bound antibodies were included as controls for all ChIP experiments. ChIP material was analysed by qPCR (Applied Biosystems 7500 real time PCR machine) using Brilliant II SYBR low ROX master mix as previously described (38) and (22) using the primers listed in Supplementary Table S3. Quantitation of target sequence enrichment in the ChIP material was calculated from the percentage of input chromatin after normalisation by subtraction of the value obtained from the no-antibody controls.

### Flow cytometry analysis

In order to quantitate levels of eGFP fluorescence following TbSAP RNAi, 1–3 × 10^6^*T. brucei* cells were washed twice in cold PSG and fixed with 2% formaldehyde. Fixed cells were then washed and resuspended in PBS. In order to determine the percentage of live or dead cells following either PFR2 or TbSAP RNAi, 0.5–1.0 × 10^6^ unfixed cells were washed and resuspended in cold PSG. Propidium iodide (PI) was added to samples to an end concentration of 10 μg/ ml with cells kept on ice to prevent internalisation of PI through endocytosis. Flow cytometry data was acquired using a BD LSR Fortessa cell analyser operated by BD FACS Diva software (BD Biosciences) with 10 000 events recorded per sample. Post-acquisition analysis of flow cytometry data was with FlowJo v10 software (BD Biosciences).

### qPCR analyses of mRNA transcript levels

Total RNA was extracted using a Qiagen RNeasy Mini kit with genomic DNA depleted using Turbo DNase (Ambion) according to manufacturer's instructions. Reverse transcription with 100 ng of total RNA was carried out using a Qiagen Omniscript RT kit with random hexamers from Promega. Generated cDNA was diluted 10-fold and the equivalent of 0.5 ng of RNA was used as the template for the qPCR reactions. qPCR reactions were carried out using an Applied Biosystems 7500 real time PCR machine using Brilliant II SYBR low ROX master mix (Agilent) with primers listed in Supplementary Table S4.

### Mass spectrometry analysis of cell surface GPI-anchored proteins

Approximately 4 × 10^7^*T. brucei* cells per sample were washed twice at 4°C in trypanosome dilution buffer (5 mM KCl, 80 mM NaCl, 1 mM MgSO_4_, 20 mM Na_2_HPO_4_, 2 mM NaH_2_PO_4_, 20 mM glucose pH 7.6) and resuspended in 45 μl cold 10 mM sodium phosphate buffer (pH 8.0) with protease inhibitors. Samples were then incubated for 5 min at 37°C in order to activate endogenous GPI-specific phospholipase C (GPI-PLC) which cleaves GPI-anchored proteins on the cell surface (39). Samples were then cooled on ice and the supernatant was harvested and diluted with NuPAGE LDS sample buffer (ThermoFisher) and dithiothreitol to a final concentration of 1× sample buffer with 100 mM dithiothreitol. Harvested proteins were denatured at 70°C for 10 min and then frozen before processing for mass spectrometry using the protocol described in (40).

### RNA-seq of bloodstream form *T. brucei* after induction of TbSAP RNAi

Total RNA was extracted using a Qiagen RNeasy Mini kit and genomic DNA was depleted using Turbo DNase (Ambion). Quality of extracted RNA was determined by agarose gel electrophoresis and TapeStation (Agilent) analysis. Generation of Illumina-indexed RNA-seq libraries and sequencing (HiSeq 4000, Illumina) was carried out at the BRC Genomics facility (Imperial College London). RNA-seq libraries were generated by polyA-enrichment for mRNA and strand selection. Paired-end sequencing was carried out to a read length of 75 bp at each end, resulting in approximately 13 million pairs of reads for each sample. Reads were processed using Trim Galore v0.4.4 (www.bioinformatics.babraham.ac.uk; -q28—illumina—stringency 3—length 50—max _n10—trim-n—paired–trim1) and aligned to reference sequence using bowtie2v2.3.4 (41) (–no-mixed—no-discordant-I50-X 500). Alignment was performed against the long-read based assembly of the *T. brucei* 427 genome (427_2018; v42). For comparative analysis to a previously published metacyclic transcriptome (42), reads from study SRP103532 (www.ebi.ac.uk/ena/) were remapped to the 427 long-read assembly with the parameters above.

Total transcript abundance was assessed using HTSeq v0.5.4 (43) considering only fragments mapping with MAPQ≥2 (to discriminate between ES copies). Differential expression was estimated using edgeR package (44), using the ‘RLE’ method for calculation of normalisation factors, an estimate of dispersion robustified against outliers, and fitting count data to a quasi-likelihood negative binomial generalised log-linear model [glmQLFit method]. To compare transcriptomic changes due to TbSAP RNAi to those in the metacyclic transcriptome, databases were remapped to the long-read assembly of the *T. brucei* 427 genome (v46 based on the work of (45)) as above. Read depth analysis was performed using bedtools v2.25.0 (46). Alternatively, the RNA-seq reads were mapped to the *T. brucei* 427 genome and expression comparison was carried out using DEseq.

## RESULTS

### A genome-scale RNAi library screen identifies a putative MES repressor protein

**Figure 1.**
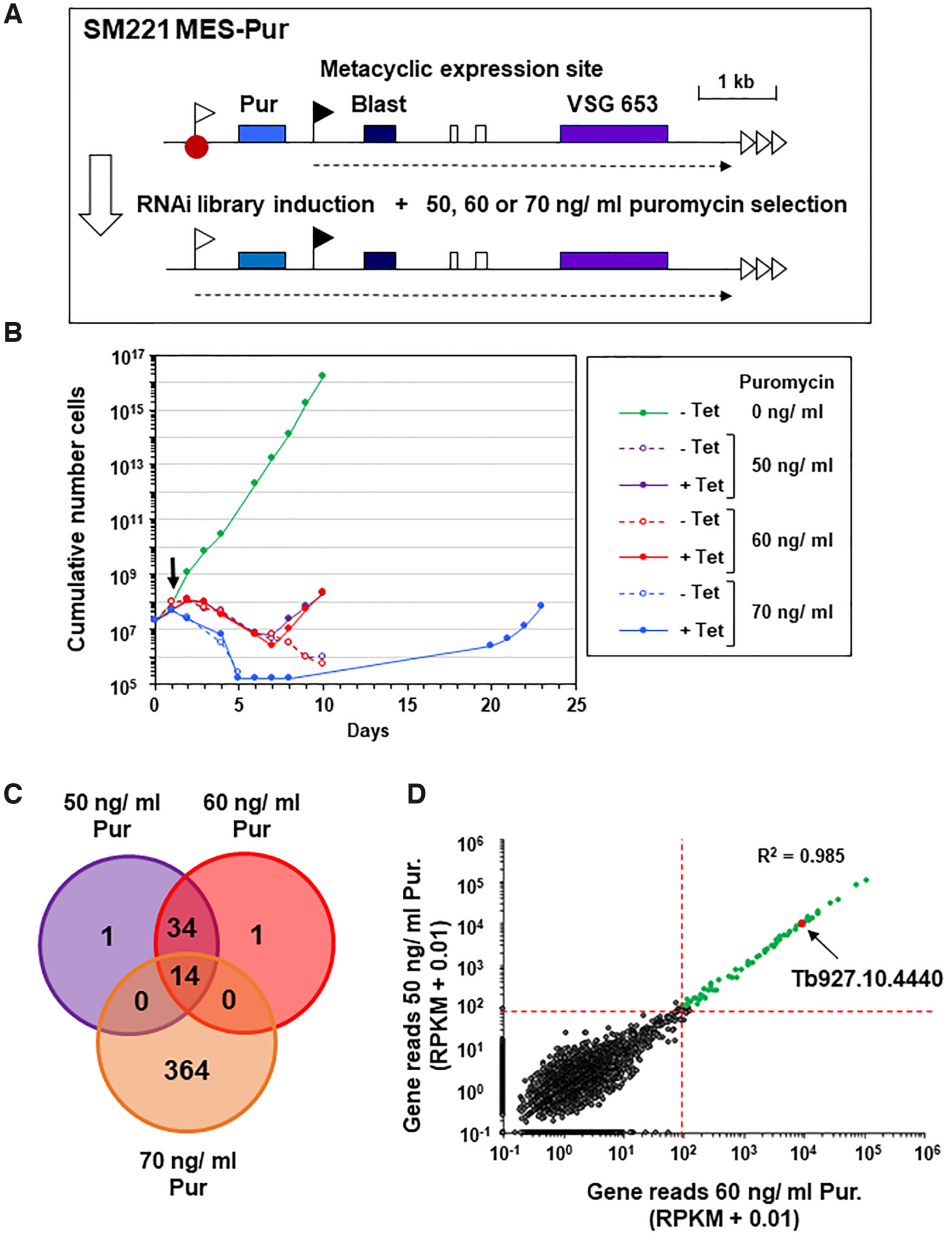
A whole genome RNAi library screen designed to identify repressors of metacyclic expression site (MES) transcription in bloodstream form *T. brucei*. (**A**) Schematic of the *T. brucei* SM221 MES-Pur cell line with a marked MES used for conducting the screen. A puromycin resistance gene (Pur) was inserted immediately downstream of the endogenous promoter (white flag) of the *VSG653* (purple box) MES. A downstream blasticidin resistance gene (Blast) expressed from an ectopic rDNA promoter (black flag) allowed selection for construct integration. A putative MES repressor protein is indicated with a red dot, various genes with coloured boxes, telomere repeats with horizontal arrows and transcription with a dashed arrow. After transfection of a tetracycline inducible whole genome RNAi library, the cell line was placed on different levels of puromycin selection to select for derepression of the inactive MES promoter after the induction of RNAi with tetracycline. (**B**) Cumulative growth curve of *T. brucei* RNAi library cells in the presence (+) or absence (−) of tetracycline (Tet) to induce RNAi in the presence of different levels of puromycin (Pur) selection. Tetracycline was added at day 0, and puromycin selection at day 1 (arrow). The growth curve shows the cumulative number of cells through time in the presence (+) or absence (−) of tetracycline and puromycin. (**C**) Venn diagram of shared MES repressor candidates identified from high throughput RITseq (100 read equivalent RPKM cutoff) from the 50, 60 and 70 ng/ml puromycin (Pur) selection screens. (**D**) Correlation plot of mapped reads from RITseq data from tetracycline induced trypanosome libraries selected with either 50 ng/ ml or 60 ng/ml puromycin (pur). Each dot represents a single *T. brucei* gene. The minimum threshold of 100 reads is marked by red dotted lines, and genes with <100 reads in both screens shown in dark grey, and genes with at least 100 reads in both screens shown in green. Correlation between the reads obtained with either the 50 or 60 ng/ml screens is high (Pearson correlation coefficient *R*^2^ = 0.985). TbSAP (Tb927.10.4440) is annotated with a red dot.

In order to identify novel repressors of the silent metacyclic *VSG* expression sites (MES) of bloodstream form *T. brucei*, we performed genome-scale RNAi library screens. These have previously successfully elucidated novel regulatory pathways in African trypanosomes (47–49). We first generated the bloodstream form *T. brucei* SM221 MES-Pur reporter strain, which expresses VSG221 from the *VSG221* bloodstream form expression site (BES1) (Figure 1A, Supplementary Figure S1A). We inserted a reporter construct containing a puromycin resistance gene immediately downstream of the inactive metacyclic *VSG* expression site (MES) *VSG653* promoter (10) (42). As we could not select for construct integration using the repressed MES *VSG653* promoter, we included an ectopic ribosomal DNA (rDNA) promoter directing expression of a blasticidin resistance gene downstream of the silent puromycin resistance gene (Figure 1A).

The MES repressor RNAi library screen involved introducing a plasmid library containing fragments of the entire *T. brucei* genome cloned between opposing tetracycline inducible T7 promoters into the *T. brucei* SM221 MES-Pur reporter strain (33). Use of the rare cutting homing endonuclease I-SceI, allowed high efficiency targeting of the plasmid library to rDNA spacers (50). Knockdown of a putative MES repressor through the induction of RNAi with tetracycline would result in derepression of the normally inactive MES *VSG653* promoter, and increased puromycin resistance of the selected trypanosomes. Readthrough transcription from the MES located ectopic rDNA promoter in the SM221 MES-Pur strain resulted in increased expression of MES *VSG653* transcript (Supplementary Figure S1B). However, introduction of the construct did not result in significantly increased transcription from the endogenous MES *VSG653* promoter, as the relative puromycin resistance of the *T. brucei* SM221 MES-Pur strain was approximately equivalent to that of the parental *T. brucei* SM221 cells (50–100 ng/ml puromycin) (Supplementary Figure S1C).

We therefore performed the MES repressor RNAi library screen three times using three different concentrations of puromycin (50, 60 and 70 ng/ml) in order to select at different degrees of stringency and thereby increase chances of success (Figure 1B). First, we electroporated the RNAi library into SM221 MES-Pur cells, generating a *T. brucei* RNAi library with an estimated 6.3x coverage of the haploid *T. brucei* genome. RNAi was induced with tetracycline at time point 0, and cells were subsequently placed on selection with puromycin 24 h later. Cells in the absence of tetracycline and puromycin (–Tet, 0 ng/ml Pur) grew at a normal rate (Figure 1B). Cells in the presence of puromycin but no tetracycline to induce RNAi died.

RNAi library induction and selection in 50 or 60 ng/ml puromycin, led to reduced growth for seven days, followed by the emergence of a resistant population. In the presence of 70 ng/ml puromycin, growth retardation was more pronounced, with a resistant population only emerging at day 19. Genomic DNA was harvested when each library had achieved a consistent robust growth rate (Figure 1B), and the remaining RNAi target fragments were amplified, using RNAi construct-specific primers (Supplementary Figure S2A).

RIT-seq (RNA interference target sequencing) was carried out to identify the RNAi target fragments enriched after puromycin selection. The PCR-amplified RNAi target fragments were deep sequenced, generating >3 million sequence reads per selected RNAi library, of which 16–24% contained the RNAi construct barcode. Barcode-containing reads were mapped against the *T. brucei* 927 reference genome and read values were converted to reads per kilobase per million reads mapped (RPKM) to account for read depth variations between the sequenced RNAi libraries. High confidence hits were identified as those represented by RPKM equivalent to >99 reads containing the RNAi construct barcode. Selection in 50 or 60 ng/ml puromycin followed a similar growth profile and identified a highly similar set of genes (Pearson's correlation coefficient of *R*^2^ = 0.985) (Figure 1C, D).

In contrast, selection in 70 ng/ml puromycin resulted in delayed emergence of a resistant population and a substantially greater number of apparent hits, which showed little concordance with those identified following selection in 50 or 60 ng/ml puromycin (Pearson's correlation coefficients of *R*^2^ = 0.104 or *R*^2^ = 0.102 respectively) (Supplementary Figure S2B, C). We initially assumed that selection in 70 ng/ml puromycin would enhance the stringency of our screen. However, the delayed recovery time and the large number of hits identified, suggests that secondary adaptations occurred in a founder population, enabling a proportion of cells to survive in the presence of puromycin, independent of specific RNAi library-mediated knockdown, thereby reducing the stringency of the screen. Hence, while selection in 70 ng/ml puromycin identified some hits also seen following selection in 50 and/or 60 ng/ml puromycin, none of the 364 unique hits were investigated further. Forty eight hits were identified following selection in 50 and 60 ng/ml puromycin, of which 14 were also identified following selection in 70 ng/ml puromycin (Figure 1C, Supplementary File S1). Remarkably, no genes were identified that had been previously documented to play a role in the repression of BESs (reviewed in (13)), suggesting that in bloodstream form *T. brucei* MES silencing is mechanistically fundamentally different to the silencing of BESs.

**Figure 2.**
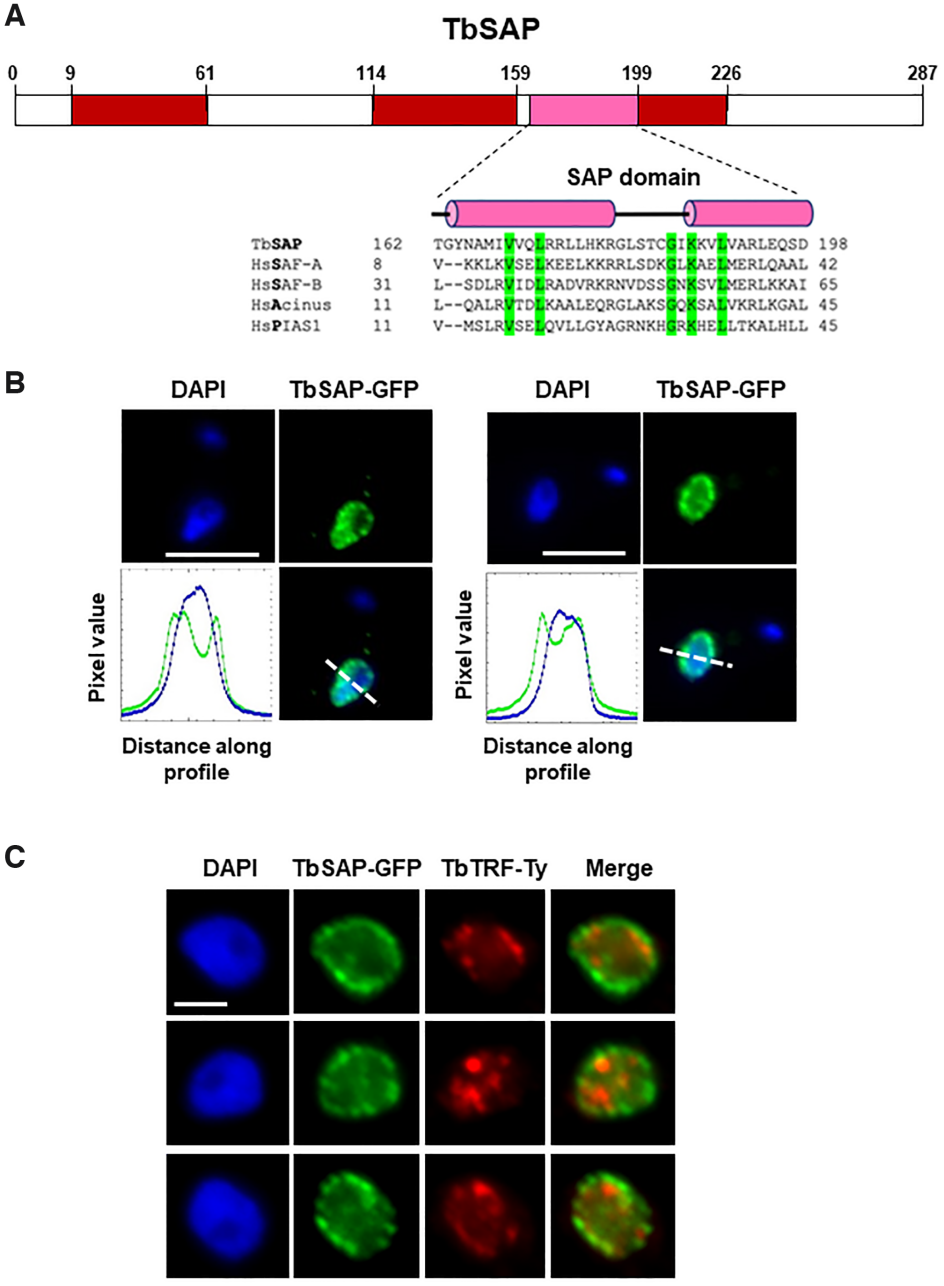
TbSAP is a protein with a canonical SAP domain which is enriched at the nuclear periphery of bloodstream form *T. brucei*. (**A**) Schematic of TbSAP (Tb927.10.4440) with a canonical SAP domain indicated with a pink box and disordered domains with red boxes as determined using InterPro. The predicted SAP domain of TbSAP is aligned with SAP motifs from *Homo sapiens* (Hs) SAF-A, SAF-B, Acinus and PIAS1 proteins using T-coffee (http://tcoffee.crg.cat/apps/tcoffee/). Conserved amino acid residues are highlighted in green, and helical domains with pink cylinders as predicted using PSIPRED. (**B**) Enrichment of TbSAP at the nuclear periphery is shown in representative nuclei of bloodstream form *T. brucei* expressing a SAP-eGFP fusion protein from the endogenous TbSAP locus. DNA was visualised with DAPI and GFP using an anti-GFP antibody. Pixel intensity of each colour along a profile (white dashed line) was calculated using the ImageJ BAR multi-plot analysis tool, with the blue line indicating DAPI stained DNA and the green line SAP-GFP. The white scale bar indicates 5 μm. (**C**) TbSAP foci do not obviously colocalise with regions enriched for the telomere binding protein TbTRF. Representative nuclei (1K1N) of a *T. brucei* cell line containing one allele of TbSAP tagged with eGFP and one allele of TbTRF tagged with a 10X Ty1 epitope are shown. SAP-GFP is visualised with an anti-GFP antibody (green), TbTRF with an anti-Ty1 antibody (red) and DNA is stained with DAPI. Scale bar represents 2 μm.

Most of the proteins identified following puromycin selection of our MES Pur RNAi library have no obvious connection with transcriptional regulation, suggesting that rather than derepressing the MES *PUR* gene expression, they may influence puromycin action (either directly or indirectly), with their depletion reducing *T. brucei* drug sensitivity. However, of the 14 hits identified in all three screens, Tb927.10.4440 stood out as the only protein with a known nuclear localisation (Tryptag.org; (51); (Supplementary File S1). This protein contains a predicted SAP DNA binding domain (InterPro) (Figure 2A), a 35 residue motif consisting of two parallel α-helices connected by an extended loop (52).

The SAP motif was first identified on the scaffold attachment factors A and B (SAF-A and SAF-B), and was later found on a range of proteins involved in nucleic acid metabolism (53). It was postulated that this SAP motif could target proteins to specific chromosomal locations. In addition, both SAP as well as the related LEM motif, were proposed to be involved in tethering chromatin to the nuclear periphery, thereby impacting on chromosomal organisation within the nucleus (52). As this, and Tb10.v4.0034 (which is identical at the amino acid level) are the only proteins in the *T. brucei* genome matching a SAP domain profile (*e* value < 1e^–3^), we named it TbSAP, and focused on it for further investigation.

### TbSAP is a nuclear protein enriched at ES promoters and MESs

We first determined the cellular localisation of TbSAP by epitope tagging the N-terminus with eGFP in bloodstream form *T. brucei* using CRISPR-Cas9 (27,54). We validated correct epitope tagging of an endogenous TbSAP gene using PCR (data not shown). As fluorescence signal from the eGFP tagged TbSAP protein was weak, we performed immunofluorescence microscopy using anti-GFP antibodies (Figure 2B). TbSAP had a heterogeneous distribution in the nucleoplasm, but was particularly enriched in foci at the nuclear periphery. These results correspond with the cellular localisation of TbSAP in procyclic form *T. brucei* documented by the TrypTag initiative (TrypTag.org).

Telomeres in bloodstream form *T. brucei* are particularly enriched at the nuclear periphery (55,56). Our attempts at combining TbSAP localisation with DNA fluorescence in situ hybridisation (FISH) to visualise *T. brucei* telomeres were unsuccessful. We therefore tested for colocalisation of TbSAP with the *T. brucei* TTAGGG-binding factor (TRF). TbTRF protein binds telomeres, and plays an important role in telomere biology, including suppressing *VSG* switching mediated by DNA rearrangements (57–59). We therefore epitope tagged TbTRF at the N-terminus with the Ty epitope in cells containing an allele of TbSAP which was endogenously tagged with eGFP. Immunofluorescence microscopy experiments visualising both TbTRF and TbSAP at the same time showed that TbSAP foci were often adjacent to TbTRF foci, but we did not see obvious colocalisation of the two proteins (Figure 2C). We do not think that the N-terminal addition of eGFP to TbSAP in bloodstream form *T. brucei* compromised its function, as knocking out the second TbSAP allele in these cells using CRISPR/Cas9 was successful (Supplementary Figure S3A). This did not alter the cellular localisation of the epitope tagged TbSAP, and as expected, nuclear staining of TbSAP was enriched at the nuclear periphery (Supplementary Figure S3B). In addition, even though tagging one allele of SAP with eGFP led to a slight reduction in growth compared with the parental cell line, this was not further reduced after the knock-out of the second SAP allele (Supplementary Figure S3C). This argues that the SAP-GFP protein was not functionally impaired to a very significant extent.

**Figure 3.**
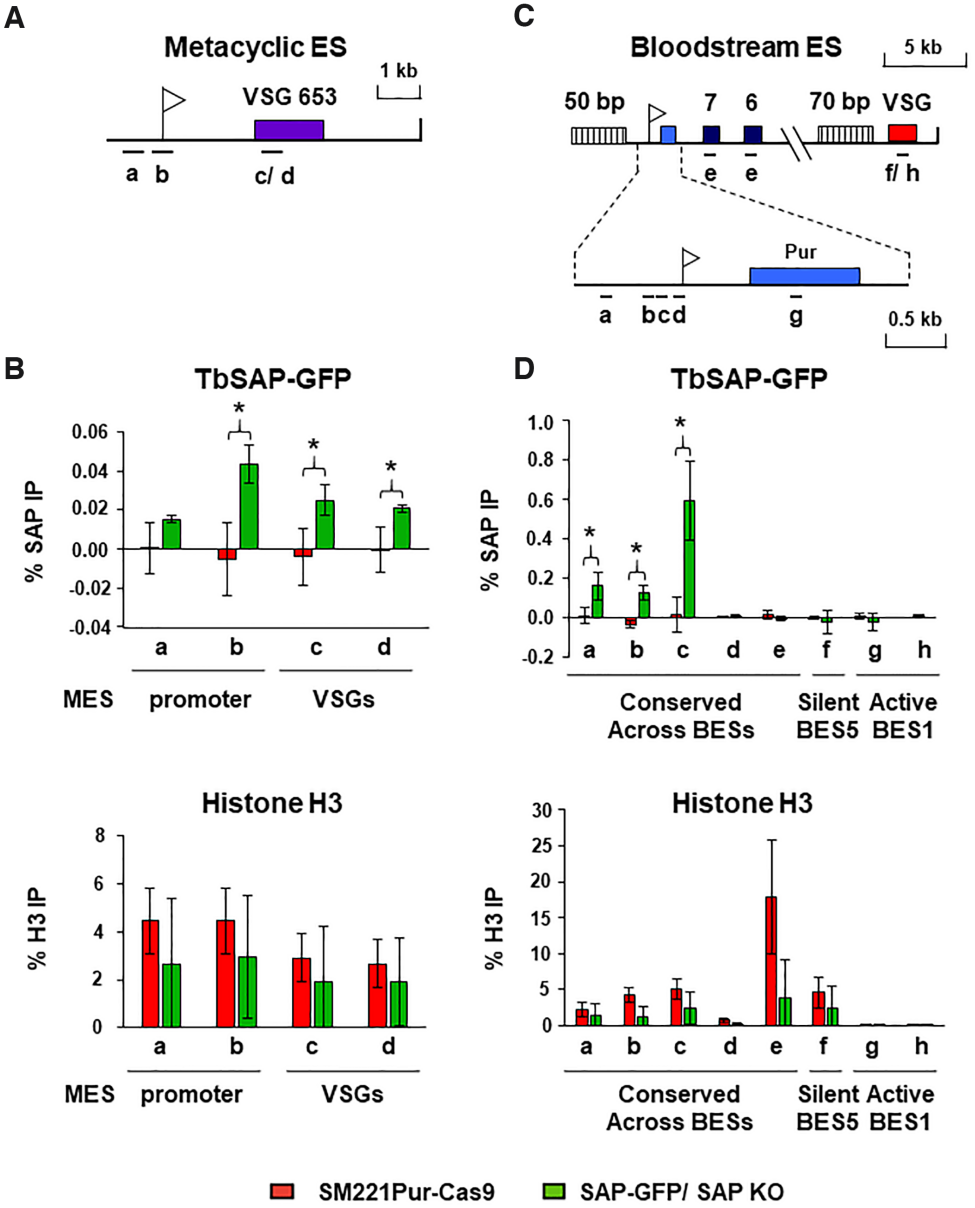
TbSAP is enriched at the promoter regions of metacyclic and bloodstream form ESs. (**A**) Schematic of a telomeric metacyclic ES (MES) with the promoter indicated with a white flag and the metacyclic *VSG653* gene with a purple box. Primer pairs used for analysis of chromatin immunoprecipitated (ChIP) material are indicated with lettered bars. Primer pair ‘c’ recognises *VSG653* and primer pair ‘d’ recognises *VSG397* present within a different MES. (**B**) The level of SAP-GFP present at MESs in the SAP-GFP/SAP KO line (green bars), which has one allele of TbSAP epitope tagged with eGFP, and the other TbSAP allele knocked out. In comparison, the same ChIP was performed with the parental cell line (*T. brucei* SM221 Pur-Cas9) (red bars) which does not contain epitope tagged TbSAP. In the top graph the ChIP was performed with an anti-GFP antibody. In the lower graph ChIP was performed using anti-histone H3 antibodies. Results are presented as the percentage of the total input immunoprecipitated after subtraction of a no antibody control. Data for the ChIP experiments is the mean of three biological replicates, with the standard deviation indicated with error bars. Statistical significance (pair-wise t-test against ChIP material from the untagged parental SM221pur cells) is indicated with asterisks (*P< 0.05). (**C**) Schematic of bloodstream form ES (BES1) with a puromycin resistance gene (blue box) inserted behind the active BES1 promoter (white flag). Expression site associated genes (ESAG) 7 and 6 are indicated with numbered boxes, and *VSG221* with a red box. Simple sequence repeats (50 or 70 bp) are indicated with vertically striped boxes. An enlargement of the BES promoter region is indicated below. Primer pairs a–e (indicated with bars) are conserved across all BESs, while primer pairs f-h are specific for sequences present within either the active BES1 (*VSG221*, primer pair ‘f’ and puromycin, primer pair ‘g’) or the silent BES5 (*VSG800* or *VSG-18*, primer pair ‘h’) respectively. (**D**) The ChIP experiments were performed as detailed in panel (**B**) only the distribution of SAP-GFP or histone H3 was determined across BESs in either the *T. brucei* SAP-GFP/ SAP KO line, or the untagged parental *T. brucei* SM221Pur-Cas9 cell line.

As the SAP motif is a DNA binding domain, we determined the distribution of TbSAP-GFP on *T. brucei* DNA using chromatin immunoprecipitation (ChIP). As TbSAP-GFP was functional, we performed the ChIP experiments in the *T. brucei* TbSAP-GFP/ TbSAP KO cells. The ChIP experiments were also performed in the parental *T. brucei* SM221pur-Cas 9 cell line as a negative control (Figure 3). Unfortunately, we were never able to isolate enough anti-GFP-TbSAP ChIP enriched DNA to perform ChIP-seq. We therefore analysed the TbSAP-ChIP enriched DNA using qPCR. We first determined the distribution of TbSAP over MES *VSG653* (Figure 3A). MES sequences are divergent from each other, and we were not able to use universal primer pairs to analyse TbSAP distribution over all MESs. However, a low but significant level of TbSAP was detected at the MES *VSG653*, with higher levels observed at the MES promoter (primer b) (*P < 0.05) (Figure 3B upper panel). TbSAP was also detected at the *VSG* gene in MES *VSG653* (primer c), as well as the *VSG* in MES *VSG397* (primer d). Histone H3 was also immunoprecipitated in parallel ChIP experiments as a control, and showed an approximately equivalent distribution in both cell lines (Figure 3B lower panel).

We next determined the distribution of TbSAP over the 15 BESs of *T. brucei* 427, which contain large stretches of highly similar DNA sequence (Figure 3C) (12). A significant enrichment of TbSAP was observed in the region immediately upstream of the BES promoters (*P < 0.05) (primers a–c in Figure 3D, upper panel), with negligible binding of TbSAP to BES downstream regions. More TbSAP appeared to be immunoprecipitated on DNA from the vicinity of BES compared with MES promoters. This is presumably as the BES promoter primers recognise ∼20 highly conserved BES promoters, compared with the MES *VSG653* primers which identify single copy sequence. Again as a control, the distribution of histone H3 was determined in both *T. brucei* cell lines, which as expected, was enriched on silent BES5 sequences and depleted on active BES2 sequences (Figure 3D, lower panel). The distribution of TbSAP over the Pol I transcribed rDNA and Pol II transcribed actin loci was also determined (Supplementary Figure S4A). There was some TbSAP observed within the 20 rDNA transcription units, with reduced levels detected in the rDNA spacers presumably reflecting the relative divergence of these sequences compared with the conserved 18S and 28S rDNA genes. Negligible amounts of TbSAP were found at the constitutively transcribed actin Pol II loci (Supplementary Figure S4B). As a control, the distribution of histone H3 was determined, which is particularly enriched in nontranscribed regions (Supplementary Figure S4C) (60).

### TbSAP knockdown leads to MES derepression in bloodstream form *T. brucei*

**Figure 4.**
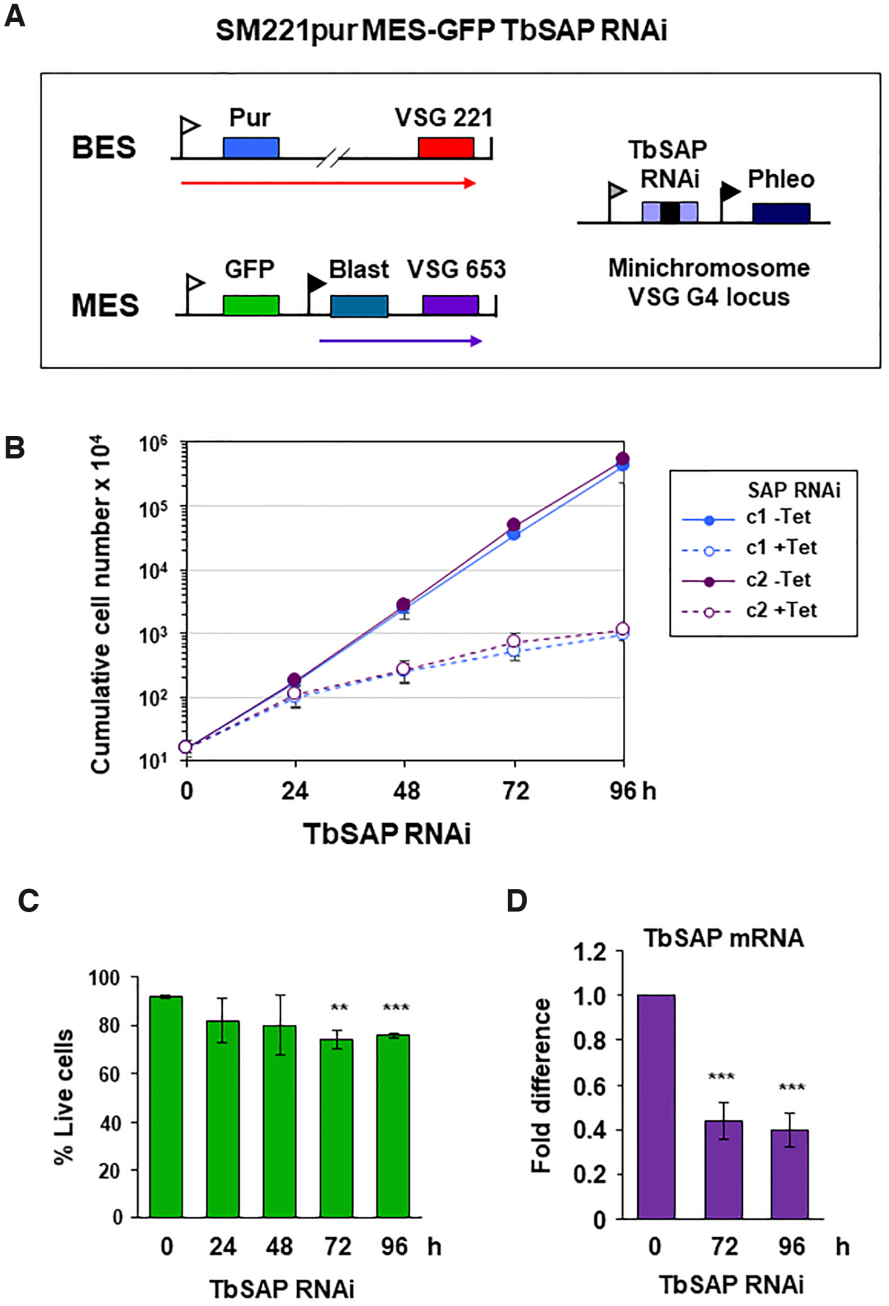
RNAi mediated depletion of TbSAP results in a reduction in growth rate with minimal lethality in bloodstream form *T. brucei*. (**A**) Schematic of the SM221pur MES-eGFP reporter cell line which expresses *VSG221* from BES1, and which is selected for with a puromycin resistance gene (Pur). An eGFP gene was inserted immediately downstream of the endogenous MES *VSG653* promoter (white flag). The construct was selected for using a downstream blasticidin gene (Blast) driven by an ectopic rDNA promoter (black flag). Transcription is indicated with arrows. A TbSAP stem-loop RNAi construct containing a tetracycline inducible T7 promoter (grey flag) was inserted into a locus containing the single copy minichromosomal *VSGG4* gene (28). (**B**) Cumulative growth curve for two MES-eGFP TbSAP RNAi clones (c1 and c2) in the presence (+) or absence (−) of tetracycline (Tet) to induce TbSAP RNAi. Time is indicated in hours (h). The mean of three biological replicates is shown with standard deviation indicated with error bars. (**C**) Determination of live/ dead bloodstream form *T. brucei* cells stained with propidium iodide and analysed by flow cytometry after the induction of TbSAP RNAi for the time indicated. Data for clone c1 are shown with error bars representing the standard deviation from three biological replicates. Tests for significance (*t*-test) show slightly increased lethality after induction of TbSAP RNAi compared with time 0, with statistically significant results indicated with **P< 0.01 and ***P< 0.001. (**D**) Quantitation of TbSAP transcript with qPCR after induction of TbSAP RNAi for the time indicated in hours (h). Data from clone c1 are shown with error bars representing the standard deviation from three biological replicates. Statistical significance for the knock-down was determined using t-tests comparing with time 0 h (***P< 0.001).

We next determined the essentiality of TbSAP, and reproducibility of MES derepression following TbSAP knockdown. We constructed a reporter cell line with an eGFP gene inserted immediately downstream of the inactive MES *VSG653* promoter (Figure 4A). Construct integration was selected for using a downstream blasticidin resistance gene driven by an ectopic rDNA promoter. Tetracycline inducible TbSAP RNAi was expressed from a construct containing a stem–loop fragment for TbSAP inserted at a single copy *VSGG4* (*VSG-31*) gene present at a *T. brucei* mini-chromosome (28). The induction of TbSAP RNAi with tetracycline resulted in a severe reduction in growth after about 24 h, but there was no evidence for a discrete cell cycle arrest (Figure 4B). There was a minor amount of cell death, but the majority of cells were alive after 96 h (Figure 4C), indicating that the knockdown of TbSAP resulted in reduced growth rate rather than acute lethality. Quantitation of TbSAP transcript using qPCR confirmed that the induction of TbSAP RNAi resulted in its reduction to ∼40% normal levels after 72 h (Figure 4D).

**Figure 5.**
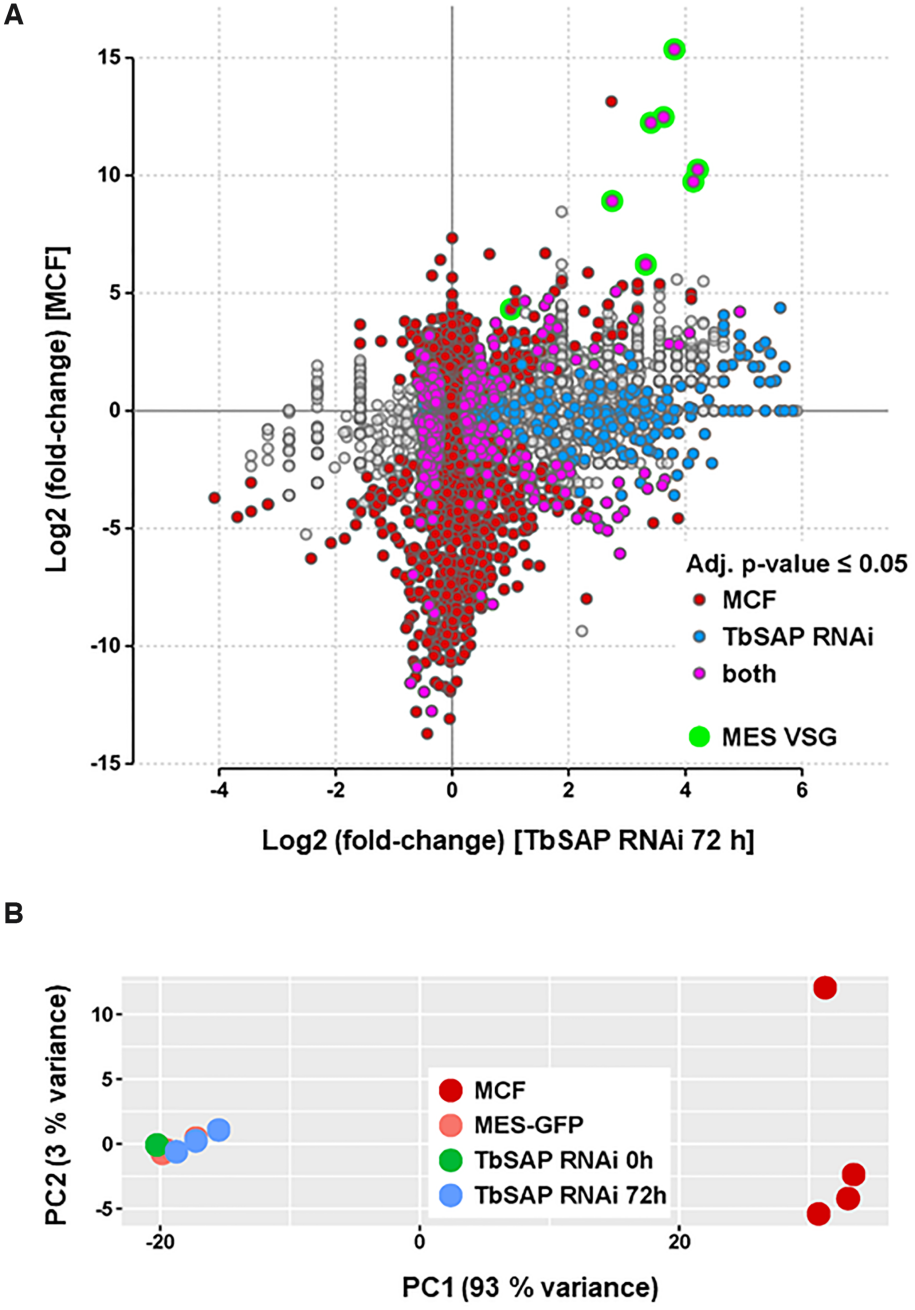
Comparison of the TbSAP RNAi transcriptome with a previously published metacyclic transcriptome reveals that although the induction of TbSAP RNAi results in the upregulation of metacyclic *VSG*s, this does not lead to ‘metacyclic-like’ cells. (**A**) Transcripts are indicated with dots, with changes in expression level after the induction of TbSAP RNAi for 72 h relative to uninduced parental cells (MES-GFP), shown on the X-axis. This is plotted against differential expression of transcripts in the metacyclic form (MCF) transcriptome described in (42) compared with parental MES-GFP cells on the Y-axis. Differentially expressed genes in either comparison (adjusted *P*-value ≤ 0.05) are highlighted. Transcripts which are only differentially expressed in the metacyclic form transcriptome are indicated in maroon. Transcripts which are only significantly changed after the induction of TbSAP RNAi for 72 h are indicated in blue. Transcripts which are significantly changed in both transcriptomes are indicated in pink, with MES *VSG*s indicated with green circles. Transcripts which have not significantly changed with respect to the transcriptome of the parental cells are indicated with grey circles. (**B**) Principal component analysis of sample to sample distances of the metacyclic form (MCF), parental (MES-GFP) or TbSAP RNAi (MES-GFP TbSAP) transcriptomes. The first two dimensions covering 96% of the variance are shown. Samples are coloured according to group.

In order to determine the effect of TbSAP knockdown on the bloodstream form *T. brucei* transcriptome, we performed RNA-seq after the induction of TbSAP RNAi for 72 h. As TbSAP was identified in a screen for MES repressors, we first investigated if we had induced a global shift to a more ‘metacyclic-like’ cell. We compared the bloodstream form transcriptome after the induction of TbSAP RNAi for 72 h with the transcriptome from the parental MES-GFP strain (X-axis of Figure 5). In addition, we compared the available transcriptome from *in vitro* derived metacyclic *T. brucei* 427 cells with the parental MES-GFP strain, which was remapped to the same assembly (Y-axis of Figure 5) (42). Transcripts which were significantly changed specifically in metacyclic forms are indicated with red dots, and those which have specifically changed after the induction of TbSAP RNAi are indicated with blue dots. Transcripts which are significantly changed in both transcriptomes compared with the parental cells are indicated with pink dots.

As expected, eight metacyclic *VSG*s were significantly upregulated in the metacyclic transcriptome (green circles) (Figure 5A). The induction of TbSAP RNAi resulted in significant derepression of seven of these different metacyclic *VSG*s (green circles). The most significantly upregulated transcripts observed both after the induction of TbSAP RNAi for 72 h in bloodstream form *T. brucei*, as well as in the metacyclic transcriptome, were metacyclic *VSG*s. However, we did not find that that knocking down TbSAP with RNAi made the cell generally more ‘metacyclic-like’, and both induced and non-induced samples cluster together distinct from the metacyclic transcriptome in principal component analysis (Figure 5B). This argues that TbSAP knockdown results in derepression of MES telomeres within cells which still fundamentally have a bloodstream form transcriptome.

**Figure 6.**
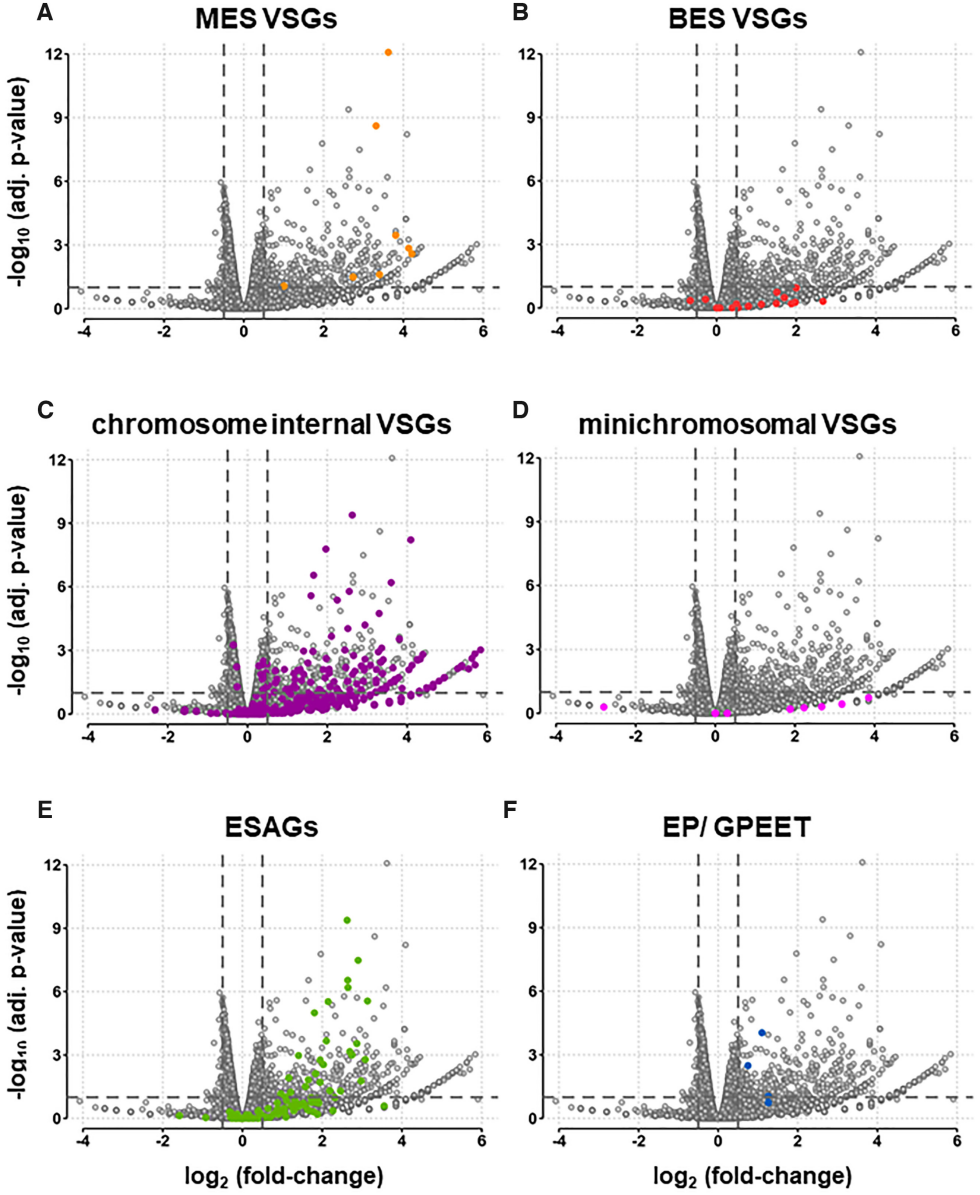
Knock-down of TbSAP leads to striking up-regulation of both *VSG*s and *ESAG*s with relatively few downregulated transcripts. (**A**) Volcano plot showing differential gene expression after induction of TbSAP RNAi for 72 h compared with the parental *T. brucei* MES-GFP cell line. Dots indicate individual transcripts with those corresponding to MES *VSG*s indicated in light orange. Results are plotted as log_2_ (fold change) of read count values versus adjusted *P*-value. Thresholds at adjusted *P*-value ≤0.1 and fold change ≥1.4 are shown (dashed lines). Values were calculated from three biological replicates of each dataset. The other panels are as in (**A**), only in panel (**B**) *VSG*s located in BESs are highlighted with dark orange dots, panel (**C**), chromosome internal (CI) *VSG*s located within the silent *VSG* arrays are highlighted with purple, panel (**D**) *VSG*s located on minichromosomes are indicated with pink dots, panel (**E**) *ESAG*s are indicated in green dots and panel (**F**) the EP and procyclin transcripts are indicated with blue dots.

When looking at the changes in the transcriptome observed after the knock-down of TbSAP for 72 h, there is a strong bias towards transcript upregulation rather than downregulation (Figure 6). In total, 397 transcripts were upregulated using a cut-off value of a 1.4-fold increase and an adjusted *P*-value of ≤ 0.1 (Supplemental file S2). In contrast, only 25 transcripts were downregulated using the same threshold. Strikingly, the degree of dysregulation was also much stronger for the genes with increased transcript abundance (mean of 6.3-fold changed for genes upregulated at fdr ≤0.1, compared to 1.2-fold for downregulated genes). This is suggestive of a predominant role for TbSAP in silencing genomic regions in *T. brucei*.

**Figure 7.**
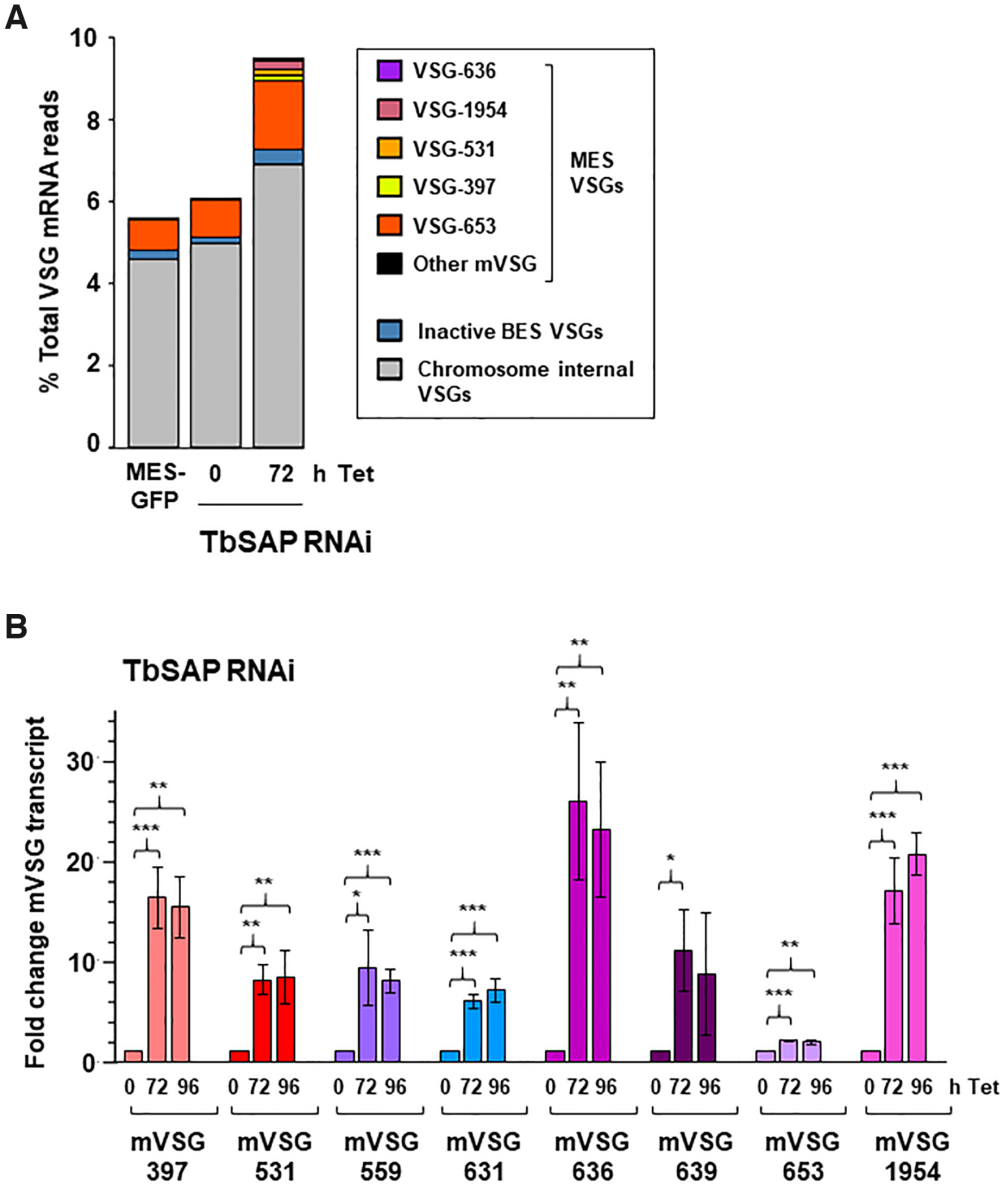
Knockdown of TbSAP results in significant upregulation of metacyclic *VSG*s in bloodstream form *T. brucei*. (**A**) Relative abundance of *VSG* transcripts following RNAi against TbSAP for 72 h. Only transcripts other than *VSG221* (*VSG-2*), present in the active BES1 are shown. The MES with *mVSG-653* is the site of integration of the reporter construct. High levels of MES *VSG*-653 expression are due to the ectopic rDNA promoter which has been introduced in the construct. Abundance is shown as a percentage of the total *VSG* transcript reads. (**B**) Depletion of TbSAP leads to significant up-regulation of all eight MES *VSG*s. RT-qPCR was performed using RNA isolated from cells where TbSAP RNAi had been induced with tetracycline (Tet) for the time indicated in hours (h). Values were normalised against actin and shown as fold-change relative to the uninduced sample. Values plotted are the mean of three independent experiments with the standard deviation indicated with error bars. Statistical significance for upregulation was determined using Student's *t*-test (*P< 0.05, **P< 0.01, ***P< 0.001).

A striking number of *VSG*s in different genomic locations were upregulated, as were transcripts from Expression Site Associated Genes (*ESAG*s) found in BESs (Figures 6, 7A, Supplemental file S2). Of eight *VSG*s in *T. brucei* 427 genome assemblies sited <4 kb downstream of a putative MES promoter, seven were upregulated between 6.5- and 18.5-fold after TbSAP knockdown (Figures 6A, 7A, Supplementary Table S5). The eighth MES *VSG* was MES *VSG653*, which is the site of integration of the reporter construct, and which was upregulated to a lesser extent. A further putative metacyclic *VSG*, *VSG-631*, is a long distance from a possible MES promoter (8.9 kb), and was not differentially expressed in the RNA-seq experiments. In contrast to *VSG*s in MESs, none of the 14 *VSG*s located in BESs, were significantly differentially expressed (Figure 6B).

Significantly, knockdown of TbSAP causes a substantial upregulation of transcripts from silent subtelomeric *VSG* arrays (chromosome internal, or CI) *VSG*s (Figure 6C). In total, 121 of 2875 CI *VSG*s (4.2%) are significantly changed at the thresholds used, compared to 230 of 14 196 non-*VSG*, non-ES genes (1.6%, *P* <10^–15^; proportions test). CI *VSG* transcripts increase from 4.6 to 5% of total *VSG* transcripts in the parental or uninduced cell lines to 6.9% after the induction of TbSAP RNAi for 72 h (Figure 7A). In contrast, none of the *VSG*s located on the *T. brucei* minichromosomes appeared to be significantly upregulated after the induction of TbSAP RNAi (Figure 7D). Although there was no significant upregulation of *VSG*s located in BESs, significant upregulation of *ESAG*s located in BESs was observed (Figure 6E).

However, a concern was that the derepression of both silent *VSG*s from the *VSG* arrays, and *ESAG*s was exclusively a secondary consequence of compromised transcription in sick cells. To resolve this, we determined the transcriptome of cells where RNAi against α-tubulin had been induced for 16 h as a lethality control. The induction of RNAi against α-tubulin results in an abrupt growth arrest, with disruption of cytoskeletal structures and cells unable to undergo cytokinesis (61). In spite of ablation of TbSAP having a much weaker effect on cell growth, more chromosomal internal *VSG*s from the *VSG* arrays (CI *VSG*) were upregulated after induction of TbSAP RNAi compared with after induction of α-tubulin RNAi (Supplementary Figure S5A). We did not find a correlation between which *VSG* transcripts were upregulated after both perturbations (Pearson's coefficient *R*^2^ = 0.0017). A similar trend was found for the *ESAG* transcripts although the degree of upregulation was less (Supplementary Figure S5B).

To confirm the derepression of MES *VSG*s seen in the RNA-seq, we analysed RNA levels by quantitative RT-PCR (qPCR) (Figure 7B). We observed that all eight *T. brucei* 427 MES *VSG*s were significantly derepressed after 72 h of TbSAP RNAi. Highest were MES *VSG636* with 25.6 ± 7.7-fold upregulation (**P ≤ 0.01), MES *VSG1954* with 16.8 ± 3.2-fold (***P ≤ 0.001) and MES *VSG397* with 16.2 ± 3.0-fold upregulation (***P ≤ 0.001) (Supplementary Table S5). The MES *VSG653* which contained the reporter construct was only upregulated 2.1 ± 0.1 fold after knockdown of TbSAP. This SM221pur MES-GFP cell line expresses relatively high levels of *VSG653* as a consequence of readthrough transcription from the exogenous rDNA promoter integrated upstream of the telomeric *VSG653* (Supplementary Figure S6A, B). These relatively high background levels possibly influence the amount of derepression observed. In agreement with the qPCR quantitation of the *VSG653* transcript, levels of fluorescence from the eGFP gene inserted upstream of the ectopic rDNA promoter within this MES also showed only 1.4–1.6-fold increase after SAP knockdown (Supplementary Figure S6C). It is likely that the genetic modification of this MES impacted its ability to be derepressed.

A concern with these MES derepression experiments, is that the upregulation of MES *VSG*s was a secondary consequence of cells responding to the reduction in cell growth rather than a phenotype specific to TbSAP. We therefore investigated if there was derepression of MES *VSG*s after knockdown of the essential gene *T. brucei PFR2* (62). As expected, induction of RNAi against PFR2 led to a very abrupt growth arrest within 8 h induction of PFR2 RNAi, with PFR2 transcript reduced to ∼30% normal levels after induction of PFR2 RNAi for 16 h (Supplementary Figure S7A–C). Despite the rapid lethality observed after knockdown of the highly essential PFR2, there was little significant increase in metacyclic *VSG* transcripts, with the possible exception of MES *VSG531* and MES *VSG1954* where we observed only a very marginal increase in transcript (Supplementary Figure S7D). This argues that the increase in MES *VSG* transcripts observed after the induction of TbSAP RNAi is indeed a specific phenotype related to TbSAP rather than a secondary effect.

### Derepression of BES promoters and chromosome internal *VSG*s after TbSAP knockdown

**Figure 8.**
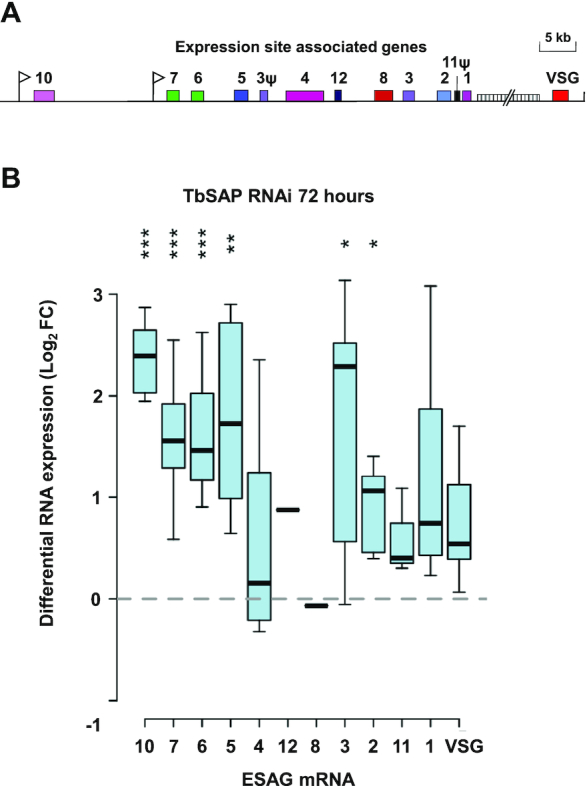
Differential upregulation of promoter proximal expression site associated genes (ESAGs) in bloodstream form expression sites (BESs) after the induction of TbSAP RNAi. (**A**) Schematic of a typical telomeric BES with promoters indicated with white flags and relevant expression site associated genes (*ESAG*s) or pseudogenes (ψ) with numbered coloured boxes (12). 70 bp repeat arrays are indicated with a vertically hatched box. (**B**) Differential regulation of *ESAG*s in inactive BESs following induction of TbSAP RNAi for 72 h. *ESAG*s have been arranged according to their common conserved position in *T. brucei* 427 BESs (12). A Tukey boxplot is shown with median (bar), interquartile range (box) and inner fences (whiskers) for all transcripts resulting from individual *ESAG*s of each family, excluding those of the active BES1. Only transcripts directed with MAPQ ≥2 at ≥1 reads per million reads are included (one transcript each of inactive *ESAG12* and *ESAG8* copies pass this threshold). Asterisks indicate Bonferroni-corrected *P*-values for individual transcript sets being different from 0 (Student's t-test; *** *P*≤ 0.001, ** *P*≤ 0.01, * *P*≤ 0.05).

**Figure 9.**
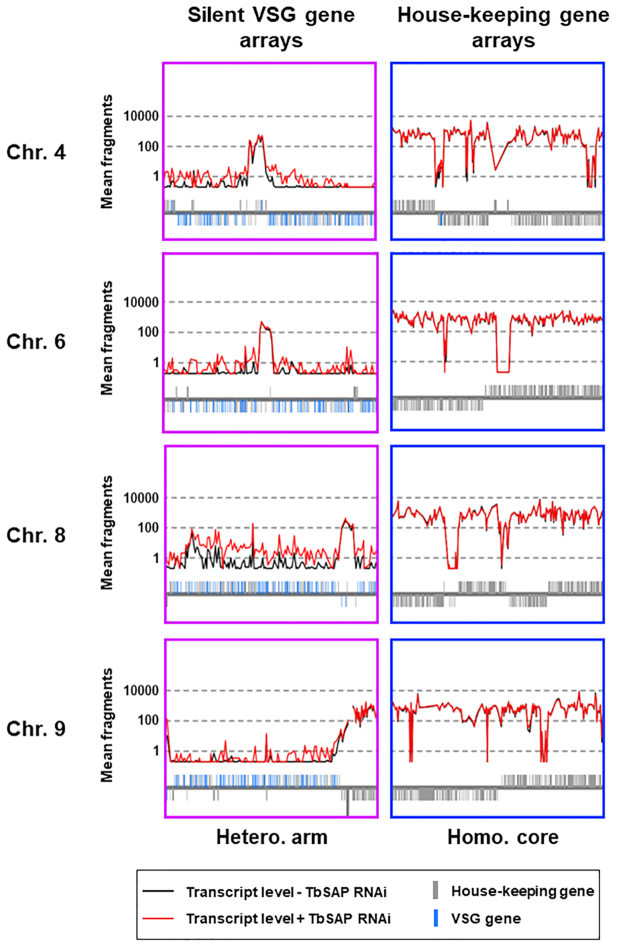
Increased transcription of silent *VSG*s present within the silent *VSG* sub-telomeric arrays after the induction of TbSAP RNAi for 72 h in MES-eGFP TbSAP RNAi cells. Transcript levels (mean fragment count over three replicates) in the presence (+) of TbSAP RNAi (red line) or absence (−) of TbSAP RNAi (black line) were mapped over the *T. brucei* megabase chromosomes. Below the traces gene architecture is shown, with housekeeping genes indicated with grey bars, and silent *VSG*s with blue bars. Magnified detail is presented from regions within chromosomes (Chr.) 4, 6, 8 and 9. These include regions within the silent *VSG* arrays present at the heterozygous transcriptionally silent aneuploid chromosomal arms (Hetero. arm). Alternatively, these regions are within the homologous transcriptionally active diploid chromosomal cores (Homo.core). In order to display on the log scale, counts of 0 have been set to 0.2).

*VSG*s at the telomeres of the BESs were not significantly derepressed after knockdown of TbSAP. However, the BES *ESAG*s, particularly those closest to the BES promoters (ESAGs 10, 7 and 6), were derepressed to a greater extent (Figure 8A, B). This indicates that although knockdown of TbSAP leads to derepression of the BES promoters, this transcription is not fully processive. We next investigated the derepressed transcription observed after TbSAP knockdown at a whole genome level (Figure 9, Supplementary Figure S8). The *T. brucei* 427 genome contains 11 diploid megabase chromosomes with extensive polycistronic transcription units containing house-keeping genes (2). After SAP knockdown, we did not see an increase in transcripts either within or at the strand-switch regions of the polycistronic Pol II transcription units (Figure 9, Homo. core).

The silent *VSG* arrays comprise one of the few non-transcribed regions in African trypanosomes, and are present as extensive single copy stretches at the arms of the diploid megabase chromosomes (45,63). In several regions of the silent *VSG* arrays, SAP knockdown led to an increase in inappropriate transcription of silent *VSG* genes (Figure 9, Hetero. arm). There are not thought to be Pol II promoters in these regions. However, it is possible that a repressive chromatin structure prevents activation of cryptic Pol II promoters (45,64). The derepression of these silent *VSG* arrays after the induction of TbSAP RNAi indicates that TbSAP could be playing a role in maintenance of a repressed chromatin state in these silenced areas of the *T. brucei* genome.

### TbSAP RNAi mediated MES derepression produces functional mVSG proteins

**Figure 10.**
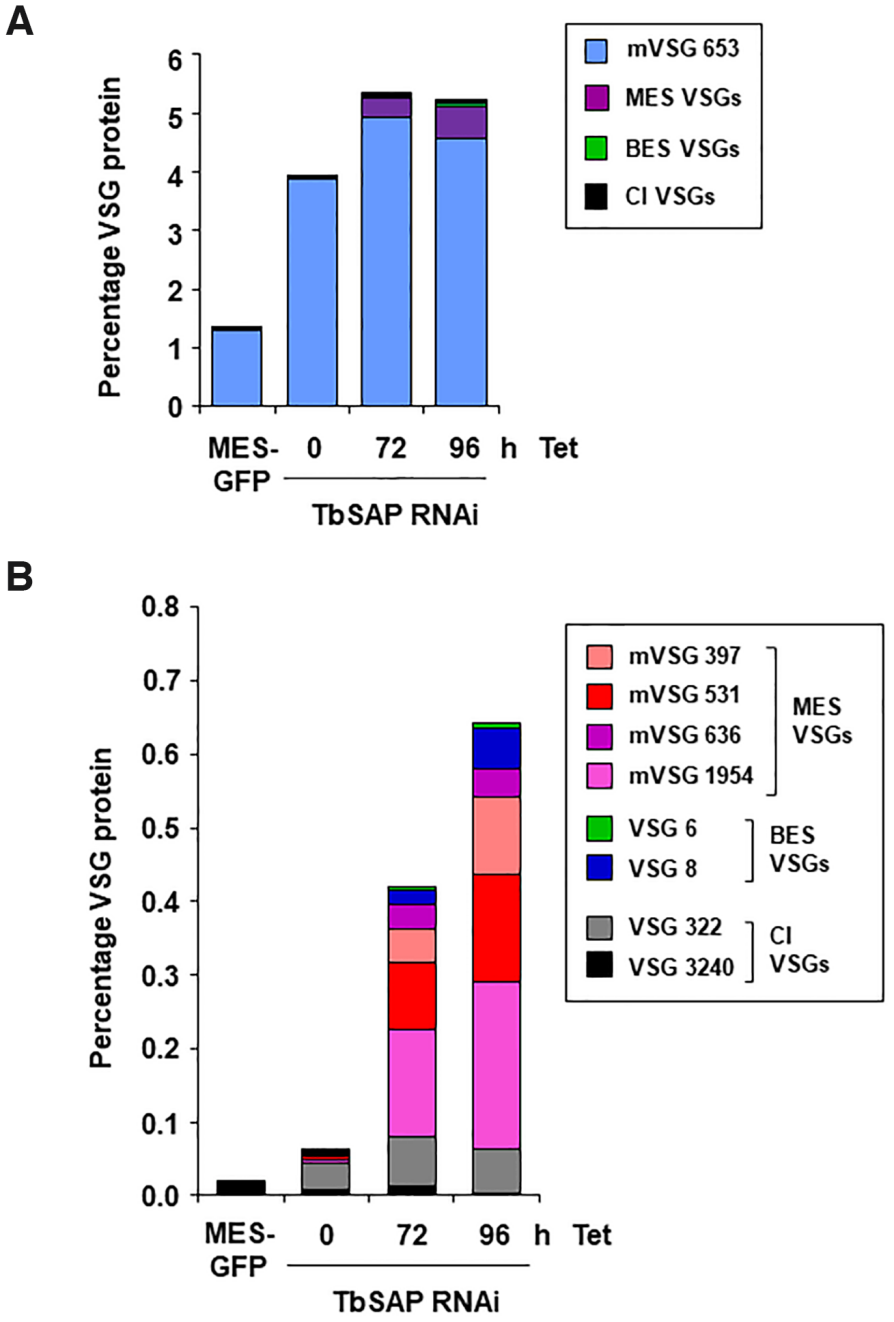
Multiple up-regulated VSGs are present on the cell surface following the induction of TbSAP RNAi (**A**) Quantitative proteomic analysis using mass spectrometry of surface VSGs cleaved by release of endogenous GPI-PLC after hypotonic lysis. Samples are from the parental VSG221 expressing SM221pur MES-GFP cells (MES-GFP) or cells where TbSAP RNAi has been induced with tetracycline (Tet) for the time indicated in hours (h). VSG221 expressed from the active BES1 is omitted from the BES VSGs dataset, as it displayed an abundance of 95–99% in each sample. In these cell lines MES VSG653 is expressed from readthrough transcription from an ectopic rDNA promoter introduced upstream of the *VSG653* gene. The different classes of VSGs are indicated with coloured bars, with MES VSG653 indicated with a blue bar, other MES VSGs (purple), BES VSGs (green bar) or VSGs in chromosome internal (CI) silent *VSG* arrays (black). Data are the mean of three biological replicates. (**B**) As in panel (A) only both MES VSG653 and VSG221 are omitted from the VSG dataset, as together they made up >99% of the sample. Different types of *VSG*s (present in either MESs or BESs) are indicated with coloured bars, with *VSG*s not located in ESs indicated as CI *VSG*s (grey and black bars). Data are the mean of three biological replicates.

We next investigated if these upregulated VSGs were present on the trypanosome cell surface, indicating functionality. Bloodstream form *T. brucei* expresses an endogenous phospholipase C (PLC) which can be activated in trypanosomes *in vitro*, where it cleaves membrane bound VSG at the GPI anchor, releasing a soluble form of VSG (39). We performed quantitative proteomics to determine the relative amounts of different VSGs in soluble VSG fractions following the induction of TbSAP RNAi. In the *T. brucei* SM221 MES-Pur cell line expressing VSG221 from the active BES1, MES VSG653 comprises 1.3% total VSG protein (Figure 10A). As mentioned earlier, this high expression level of VSG653 is presumably a consequence of readthrough from the ectopic rDNA promoter (Supplementary Figure S1). If a TbSAP RNAi construct is introduced into this cell line, the amount of MES VSG653 increases about three fold (presumably as a consequence of leaky transcription of the RNAi fragment), and then further increases after the induction of TbSAP RNAi (Figure 10A).

For the other MES VSGs, the proteomic analyses globally reflect what was observed at the transcript level. Metacyclic *VSG*s with the highest transcript reads (*VSG397*, *VSG531*, *VSG636* and *VSG1954*) were all detected by mass spectrometry following phospholipase-C cleavage, indicating localisation on the cell surface (Figure 10B). Although metacyclic VSGs are not detectable on the surface of the parental VSG221 cells, after the induction of TbSAP RNAi for 72 or 96 h, they comprise 0.35% or 0.54% of the total VSG protein respectively. Less protein was detected from *VSG*s present in either the chromosome internal *VSG* arrays or BESs than would be expected from the transcript reads. These results show that if MESs are derepressed, they can produce functional VSG protein in bloodstream form *T. brucei*.

## DISCUSSION

Recently progress has been made in identifying factors involved in BES repression in bloodstream form *T. brucei* including a range of chromatin proteins and remodelers (13). In addition, VEX1 was identified using whole genome RNAi screens, and VEX2/CAF1B through determining the VEX1 interactome (65,66). However, it is still not understood how all MESs are kept transcriptionally silent in bloodstream form *T. brucei*.

Here, using whole genome RNAi library screens, we identified a novel SAP DNA binding domain containing protein (TbSAP) which plays an important role in MES repression in bloodstream form *T. brucei*. We found that TbSAP is enriched at the nuclear periphery of bloodstream form trypanosomes, and using ChIP, established that TbSAP binds both MESs and the immediate region upstream of the BES promoters. Knockdown of TbSAP resulted in significant upregulation (up to 26-fold) of the silent MESs. However, these bloodstream form cells depleted of TbSAP retained a bloodstream form transcriptome rather than becoming ‘metacyclic’-like, indicating that TbSAP was not affecting *T. brucei* differentiation. Strikingly after knockdown of TbSAP, most of the transcripts with changed levels of expression were upregulated (397 transcripts) rather than downregulated (25 transcripts). The upregulated transcripts included *VSG*s within the inactive chromosome internal subtelomeric *VSG* arrays, which are not within transcription units. TbSAP knockdown additionally led to upregulation of *ESAGs* within the immediate vicinity of the silent BES promoters. All of these data argue that in bloodstream form *T. brucei*, TbSAP could be a repressive chromatin protein, which plays a key role in maintaining a transcriptionally inactive state at genomic regions containing silent *VSG*s.

Knockdown of TbSAP resulted in derepression of both MES and BES promoters, despite the fact that BES promoters are 30–60 kb upstream of the chromosome end compared with MES promoters which are normally in the immediate proximity (1–2 kb) of the telomeric *VSG* (10,12). However, derepression of BES promoters predominantly resulted in only limited transcription immediately downstream of the promoters comparable to what was observed after knocking down the ISWI and FACT complexes (67,68) or the histone chaperones ASF1A/ CAF1B (69). It is likely that derepressed elongating RNA polymerases are hindered by a RAP1 mediated silencing gradient extending upwards from the inactive BES telomeres of bloodstream form *T. brucei* preventing fully processive transcription (70,71).

Although knockdown of TbSAP led to some derepression of BES promoters, our RNAi library screens selecting for MES repressors did not identify any of the known BES repressors (reviewed in (13)). This argues that repression of MESs is mechanistically different from the repression of inactive BESs in bloodstream form *T. brucei*. The immediate vicinity of the BES telomere (within several kb of the telomere repeats) is more stringently silenced than upstream subtelomeric regions in bloodstream form *T. brucei* (72). As the MES transcription units are normally within 5 kb of the telomere end, it is possible that they are all kept repressed by this localised telomeric silencing gradient in bloodstream form *T. brucei*. In contrast, BES promoters, which are normally 30–60 kb upstream of the telomere repeats, would be expected to be out of range of this localised telomeric repression. It would be interesting to determine the nature of the telomeric silencing gradients operating in metacyclic *T. brucei*, where MES monoallelic exclusion operates, and only one MES escapes silencing in any given cell.

TbSAP is the only SAP domain containing protein identifiable in the *T brucei* genome. The SAP DNA binding domain comprises a two-helical bundle which was first found on the scaffold attachment proteins SAF-A and SAF-B, which bind chromosomal nuclear scaffold attachment regions (SARs) (73,74). SARs are discrete AT-rich DNA elements, which are thought to allow compartmentalisation of the eukaryotic genome into different regions of chromatin through chromosomal attachment to the nuclear scaffold. The SAP DNA binding motif was subsequently found on nuclear proteins with diverse roles including transcription, DNA repair and RNA processing. The broad range of functions of these SAP domain containing proteins, suggest that the SAP domain along with other elements present in each of these proteins may dictate their distinct functionality.

Both the SAP domain found in unicellular and multicellular eukaryotes, and the highly related LEM motif found exclusively in metazoans, are present on proteins tethering chromatin to the nuclear lamina (52). Compatible with a role in silencing telomeres, TbSAP was enriched at the nuclear periphery, possibly sequestering genomic areas containing silent *VSG*s to this region of low transcriptional activity. Although the heterogeneous distribution of SAP at the nuclear periphery was reminiscent of telomeric clusters, we did not see obvious colocalisation of TbSAP with the *T. brucei* telomere binding protein TbTRF. In addition, we did not find evidence that knockdown of TbSAP led to redistribution of *T. brucei* telomeres within the nucleus, as investigated using telomere DNA-FISH. However, most of the telomeric signal in these experiments was presumably derived from the telomeres of the ∼100 mini-chromosomes, which distribute differently to the 11 diploid megabase chromosomes (55,75). It remains possible that TbSAP specifically tethers MES and BES containing telomeres to the nuclear periphery, however, this remains to be investigated.

RNA-seq following TbSAP knockdown with RNAi revealed that BES derepression occurred primarily in promoter proximal regions. This corresponds with the ChIP experiments showing association of TbSAP with both MES and BES promoters. In addition, we found that transcripts from *VSG*s within the silent subtelomeric *VSG* arrays were upregulated. The *T. brucei* genome appears to be compartmentalised into different chromatin domains. Using Hi-C chromosome conformation capture approaches, the Siegel lab has argued that the *T. brucei* genome is partitioned within the nucleus, with the silent subtelomeric *VSG* arrays folded into distinct highly compact regions (45). While silent BESs appear to be enriched at the nuclear periphery in bloodstream form *T. brucei*, the active BES appears to be internal in the nucleus. This cellular localisation of silent BESs appears to affect transcriptional control. Knockdown of the *T. brucei* NUP1 and NUP2 proteins, which associate with the nuclear periphery, affects transcriptional repression of ESs as well as the localisation of telomeres (76,77). It is possible that TbSAP has a similar effect on inactive ES telomeres. The observation that TbSAP is present in foci, which are adjacent to, but not colocalised with TbTRF foci at the nuclear periphery, would be compatible with enriched binding of TbSAP to these condensed subtelomeric regions of the genome.

The silent *VSG* arrays, which are located at the subtelomeres of megabase chromosomes, contain thousands of silent *VSG*s, (45). Additionally, 100–200 *VSG*s are located at the telomeres of minichromosomes, which are primarily comprised of palindromic arrays of 177 bp simple sequence repeat, and have a different nuclear distribution as well as segregating differently to megabase chromosomes (55,75,78). Knockdown of TbSAP resulted in increased transcript levels of *VSG*s present within the silent *VSG* arrays but not the *VSG*s at minichromosomes. Whole genome nucleosome positioning analyses, have shown that the silent *VSG* arrays do not have precisely positioned nucleosomes, but are flanked upstream and downstream by extensive regions of chromatin with well positioned nucleosomes (64). These bordering regions of chromatin potentially prevent readthrough from upstream Pol II transcription units into the silent *VSG* arrays. In addition, the silent *VSG* arrays are likely to have more compact chromatin than the core regions of the housekeeping chromosomes, which would prevent fortuitous transcription initiation from cryptic Pol II promoters (45). It is possible that TbSAP functions as a repressive chromatin protein suppressing activation of cryptic promoter sequences, and unwanted transcription of the many thousands of silent subtelomeric *VSG* genes. The lack of derepression of minichromosomal *VSG*s upon TbSAP knockdown could be a consequence of the simple sequence structure of the minichromosomes. As they are primarily comprised of simple sequence repeats, this would presumably restrict the number of regions potentially functioning as cryptic promoters if the chromatin state of these minichromosomes became less repressive.

TbSAP was recently identified in procyclic *T. brucei* as one of several interacting partners of SNF2PH, a SUMOylated transcription factor enriched at the telomere of the active BES in bloodstream form *T. brucei* (Appendix Table S2 in (79)). In addition to TbSAP, other SNF2PH interacting partners identified included transcription regulating factors including Spt16 of the FACT complex and the Pol I transcription factor CITFA-4 (68,80). The functional significance of this association of TbSAP with SNF2PH is unclear, especially as these results were obtained in procyclic form *T. brucei*, which was not studied here. However, it is compatible with the observation that both proteins bind upstream of BES promoters as seen using ChIP experiments in this study and in Figure 3 in (79).

In conclusion, TbSAP appears to be a DNA binding protein suppressing transcription of MESs and BESs as well as *VSG*s at the silent *VSG* arrays in bloodstream form *T. brucei*. The enrichment of TbSAP at the nuclear periphery combined with the RNA-seq and ChIP data are consistent with a model whereby association of TbSAP with the promoters of silent ESs at the nuclear periphery enables transcriptional suppression. Whether this is mediated through regulation of gene localisation, or through changes in global chromatin architecture in these regions is still unclear. In agreement with a proposed global silencing function, knockdown of TbSAP led to significant upregulation rather than downregulation. TbSAP knockdown led to significant derepression of MESs, but the derepressed cells retained a bloodstream form transcriptome. This novel factor therefore gives us additional insight into one of the layers of control allowing bloodstream form *T. brucei* to keep the silent MESs as well as the many thousands of silent *VSG*s transcriptionally inactive. In addition, it gives us insight into how repressed genomic regions are kept silent in an early branching eukaryote, where most of the genome is transcriptionally active.

## DATA AVAILABILITY

Data from the RIT-Seq analyses have been deposited in the European Nucleotide Archive (ENA) at EMBL-EBI under project accession number PRJEB41048 (https://www.ebi.ac.uk/ena/browser/view/PRJEB41048). Data from the RNA-seq experiments can be accessed in the NCBI GEO repository under project number GSE160713 (https://www.ncbi.nlm.nih.gov/geo/query/acc.cgi?acc=GSE160713). Data from the proteomics analyses is available in the PRIDE database with ProteomeXchange accession number: PXD022397 (http://www.ebi.ac.uk/pride/archive/projects/PXD022397).

## Supplementary Material

gkab109_Supplemental_Files
