# Supplementary material for: TbSAP is a novel chromatin protein repressing metacyclic variant surface glycoprotein expression sites in bloodstream form Trypanosoma brucei
Source: Nucleic Acids Res. 2021 Feb 28;49(6):3242–62. doi: 10.1093/nar/gkab109 (PMC8034637; doi:10.1093/nar/gkab109)
Supplement: gkab109_Supplemental_Files [file gkab109_supplemental_files.zip › Supplemental Material SAP is a novel chromatin protein.pdf]

Supplemental Material:

TbSAP is a novel chromatin protein repressing metacyclic Variant Surface Glycoprotein expression sites in bloodstream form *Trypanosoma brucei*

Carys Davies[1], Cher-Pheng Ooi[1], Georgios Sioutas[1], Belinda S. Hall[1], Haneesh Sidhu[1], Falk Butter[2], Sam Alsford[3], Bill Wickstead[4] and Gloria Rudenko[1*]

Supplementary Figures 1-8 plus figure legends

Supplementary Tables 1-5

Figure legends Supplementary files 1 and 2.

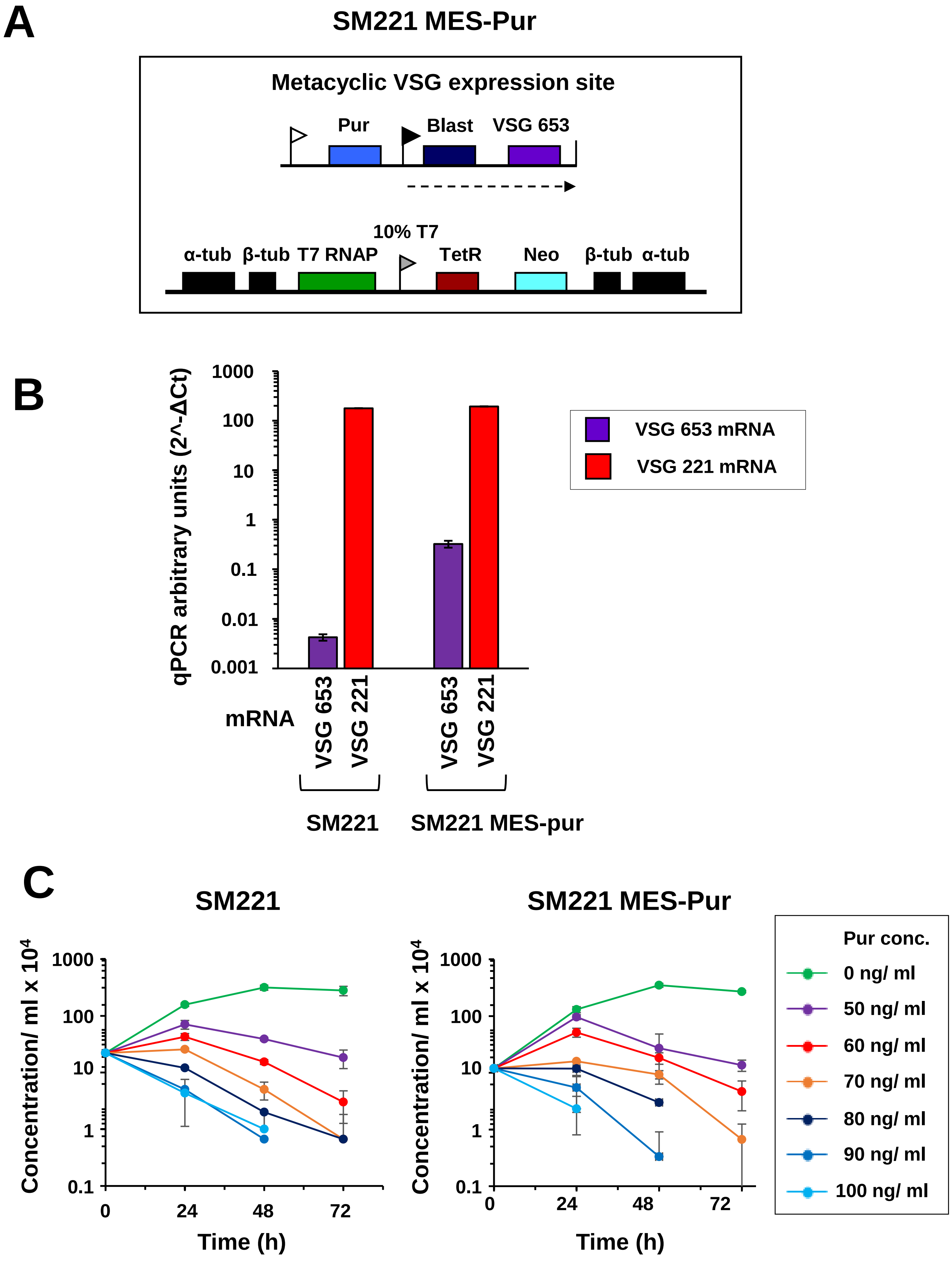
A
SM221 MES-Pur
Metacyclic VSG expression site
Pur
Blast
VSG 653
10% T7
α-tub
β-tub
T7 RNAP
TetR
Neo
β-tub
α-tub
B
qPCR arbitrary units (2^-ΔCt)
1000
100
10
1
0.1
0.01
0.001
VSG 653 mRNA
VSG 221 mRNA
mRNA
VSG 653
VSG 221
VSG 653
VSG 221
SM221
SM221 MES-pur
C
SM221
SM221 MES-Pur
Concentration/ ml x 10^4
1000
100
10
1
0.1
0
24
48
72
Time (h)
Pur conc.
0 ng/ ml
50 ng/ ml
60 ng/ ml
70 ng/ ml
80 ng/ ml
90 ng/ ml
100 ng/ ml

Sup. Fig. S1

Supplementary Figure S1

Cell line with a construct containing a puromycin resistance gene inserted behind an inactive endogenous metacyclic expression site (MES) promoter allows RNAi library screens for repressors of MES expression.

(**A**) Schematic representation of the bloodstream form VSG221 expressing SM221 MES-Pur cell line generated by integration of a construct containing a puromycin (Pur) resistance gene behind the inactive endogenous promoter (white flag) of the metacyclic expression site (MES) *VSG653*. The construct was selected for using a downstream blasticidin resistance gene (Blast) under the control of an ectopic rDNA promoter (black flag). These cells also contain a construct containing a T7 RNA polymerase (T7 RNAP) gene, a tetracycline repressor (TetR) gene under the control of a 10% T7 promoter, and a neomycin resistance gene (Neo) inserted into the tubulin locus containing α and β tubulin (tub) repeats. Relevant genes are indicated with coloured boxes.

(**B**) Quantitation of *VSG* transcript with RT-qPCR analysis from the parental (SM221) or reporter (SM221 MES-Pur) cell lines. Transcript amount from *VSG221* is indicated with red bars, and transcript amount from *VSG653* with blue bars. Error bars indicate the standard deviation from three biological replicates.

(**C**) Growth curve of the parental (SM221) or the reporter (SM221 MES-pur) cell lines after selection on different concentrations of puromycin (Pur) for the time indicated in hours (h). Error bars indicate the standard deviation from three biological replicates.

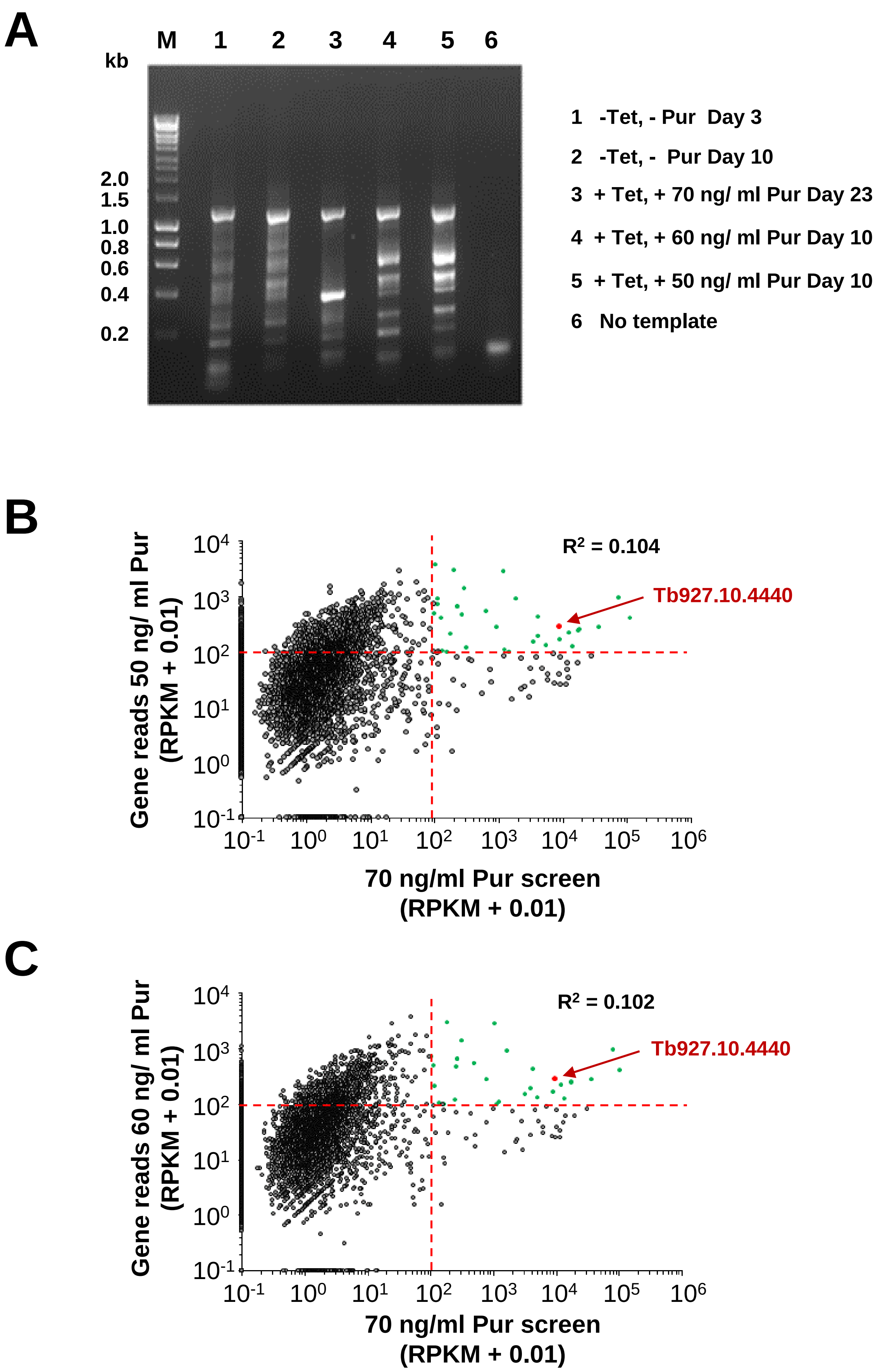
A
M 1 2 3 4 5 6
kb
2.0
1.5
1.0
0.8
0.6
0.4
0.2
1 -Tet, - Pur Day 3
2 -Tet, - Pur Day 10
3 + Tet, + 70 ng/ ml Pur Day 23
4 + Tet, + 60 ng/ ml Pur Day 10
5 + Tet, + 50 ng/ ml Pur Day 10
6 No template
B
R² = 0.104
Tb927.10.4440
Gene reads 50 ng/ ml Pur (RPKM + 0.01)
70 ng/ml Pur screen (RPKM + 0.01)
C
R² = 0.102
Tb927.10.4440
Gene reads 60 ng/ ml Pur (RPKM + 0.01)
70 ng/ml Pur screen (RPKM + 0.01)

Sup. Fig. S2

Supplementary Figure S2

Identification of putative repressors of MES transcription isolated from RNAi library screens using PCR and RIT-Seq.

(**A**) RNAi target fragments were PCR amplified from genomic DNA harvested from different MES repressor RNAi library screens. *T. brucei* was grown in the presence (+) or absence (-) of tetracycline (Tet) to induce RNAi. Genomic DNA was isolated at day 3 or 10 from trypanosomes grown without tetracycline or puromycin (Pur). DNA was isolated on day 10 for trypanosomes selected on 50 or 60 ng/ ml puromycin or day 23 for cells selected with 70 ng/ ml puromycin. The LIB2f and LIB2r primers were used for amplification of RNAi plasmid inserts as described in (34). An agarose gel of the PCR products is shown, with DNA size markers in the lane M, and sizes indicated in kilobases (kb). Sequencing the PCR products allowed the identification of TbSAP (Tb927.10.4440), which was identified in each screen.

(**B**) Correlation graph of high throughput RITseq reads obtained from the 50 ng/ ml puromycin RNAi library screen compared with the 70 ng/ ml puromycin RNAi library screens. Correlation between the two RITseq profiles is low (Pearson's correlation coefficient R^2=0.104). TbSAP (Tb927.10.4440) is indicated in red.

(**C**) Correlation graph of high throughput RITseq reads obtained from the 60 ng/ ml puromycin compared with the 70 ng/ ml puromycin RNAi library screens. Correlation between the two RITseq profiles is low (R^2=0.102). TbSAP protein is indicated with a red dot.

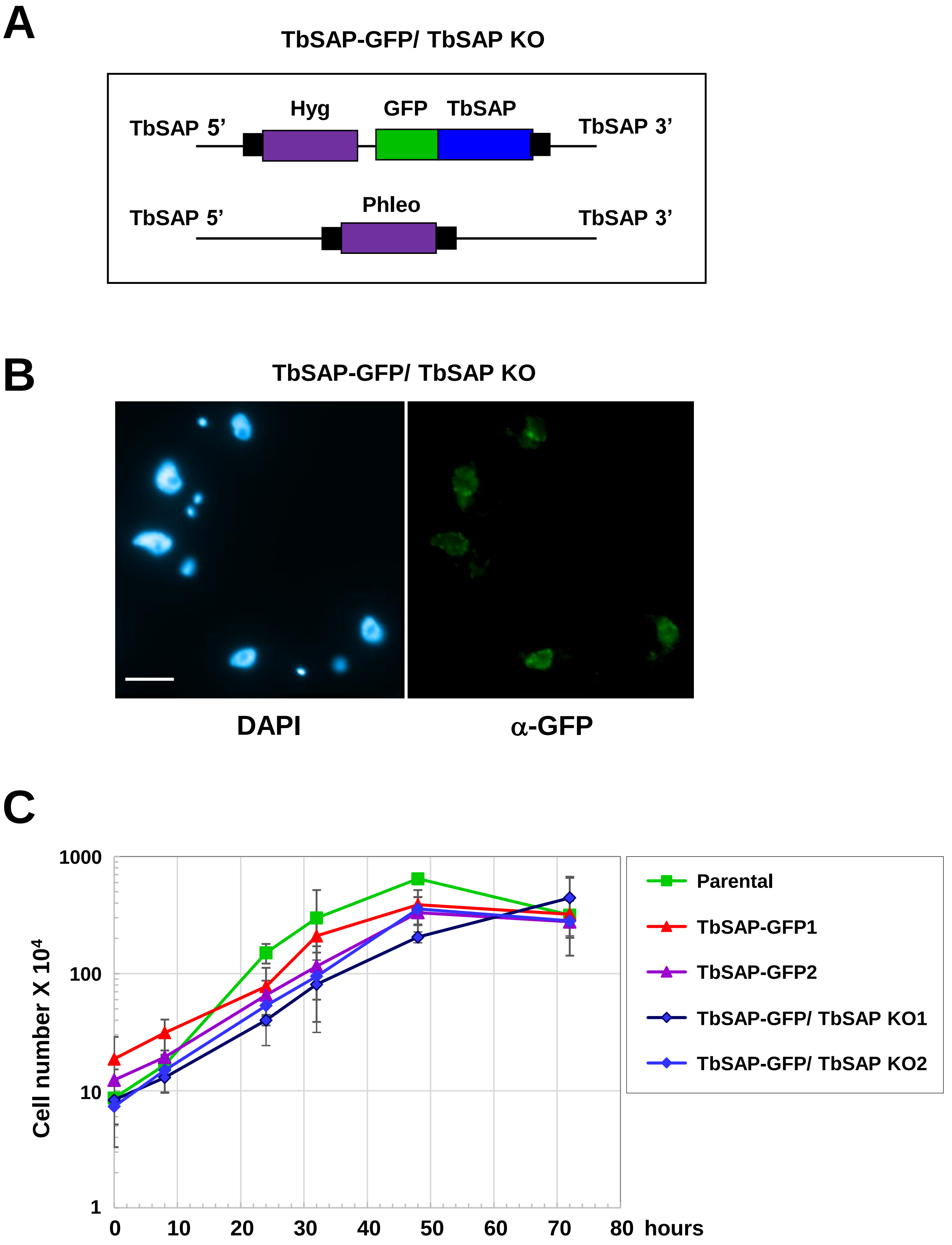
A
TbSAP-GFP/ TbSAP KO
Hyg
GFP
TbSAP
TbSAP 5'
TbSAP 3'
Phleo
TbSAP 5'
TbSAP 3'
B
TbSAP-GFP/ TbSAP KO
DAPI
α-GFP
C
Cell number X 10⁴
1000
100
10
1
0
10
20
30
40
50
60
70
80
hours
Parental
TbSAP-GFP1
TbSAP-GFP2
TbSAP-GFP/ TbSAP KO1
TbSAP-GFP/ TbSAP KO2

Sup. Fig. S3

Supplementary Figure S3

Characterisation of the *T. brucei* cell line with one allele of TbSAP epitope tagged with eGFP and the other allele knocked-out (KO).

(**A**) Schematic of the bloodstream form *T. brucei* TbSAP-eGFP/ TbSAP KO cell line. A construct was inserted into the TbSAP locus resulting in TbSAP epitope tagged at the N-terminus with eGFP. Upstream is a hygromycin (hyg) resistance gene used for selection. The second allele of TbSAP was replaced with the phleomycin (phleo) resistance gene.

(**B**) Representative immunofluorescence microscopy images of TbSAP-eGFP/ TbSAPKO cells stained with anti-GFP antibodies. The expected enrichment at the nuclear periphery is observed. On the left are the nuclei stained with the DNA stain DAPI. The scale bar represents 5 μm.

(**C**) Growth curves monitoring the rate of growth of two cell lines where one allele of TbSAP was endogenously tagged with eGFP (TbSAP-GFP1 and TbSAP-GFP2). In addition, growth curves were performed with the parental cell line, or two cell lines where one TbSAP allele had been tagged with eGFP, and the second TbSAP allele had been subsequently knocked out (TbSAP-GFP/ TbSAPKO1 and TbSAP-GFP/ TbSAPKO2).

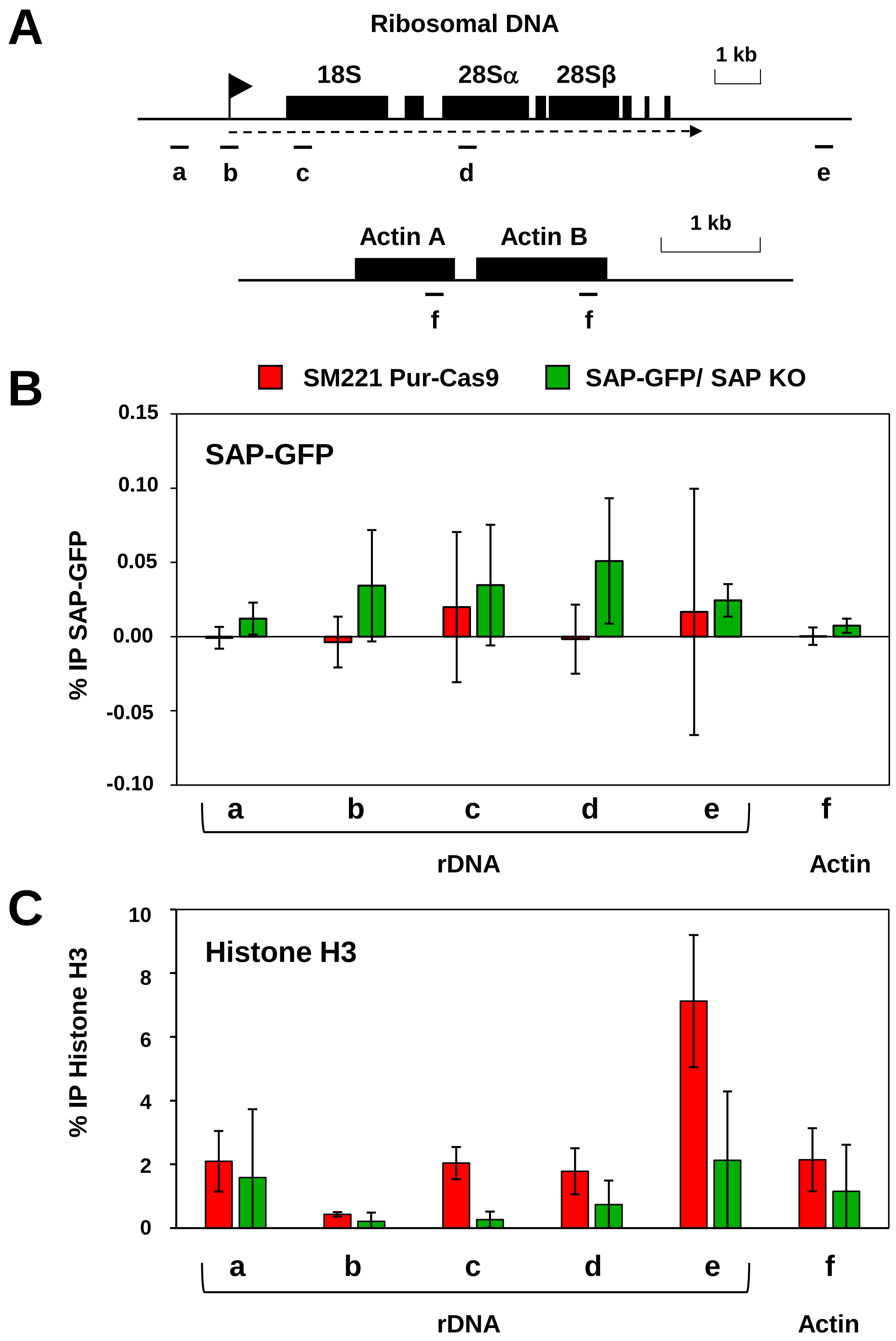
A
Ribosomal DNA
18S
28Sα
28Sβ
1 kb
a
b
c
d
e
Actin A
Actin B
1 kb
f
f
B
SM221 Pur-Cas9
SAP-GFP/ SAP KO
SAP-GFP
% IP SAP-GFP
0.15
0.10
0.05
0.00
-0.05
-0.10
a
b
c
d
e
f
rDNA
Actin
C
Histone H3
% IP Histone H3
10
8
6
4
2
0
a
b
c
d
e
f
rDNA
Actin

Sup. Fig. S4

Supplementary Figure S4

TbSAP is found on the Pol I transcribed ribosomal DNA (rDNA) locus but is not detectable at high levels at the Pol II transcribed actin locus.

(**A**) Schematic showing an rDNA transcription unit with the promoter indicated with a black flag, the rDNA genes with black boxes and transcription with a dashed arrow. Below is an actin transcription unit. Primer pairs used for qPCR quantitation of ChIP DNA are indicated with lettered bars.

(**B**) The level of TbSAP enrichment determined by ChIP-qPCR in the parental SM221Pur-Cas9 (red bars) or SAP-eGFP/ SAPKO cell lines (green bars). Results are presented as the percentage of the total input immunoprecipitated after subtraction of a no antibody control. Data for the SM221Pur-Cas9 and SAP-GFP/ SAP KO lines are the means of three independent ChIP experiments with standard deviation indicated with error bars.

(**C**) As described in panel (**B**), only the level of histone H3 was determined over the same genomic regions in both cell lines.

A

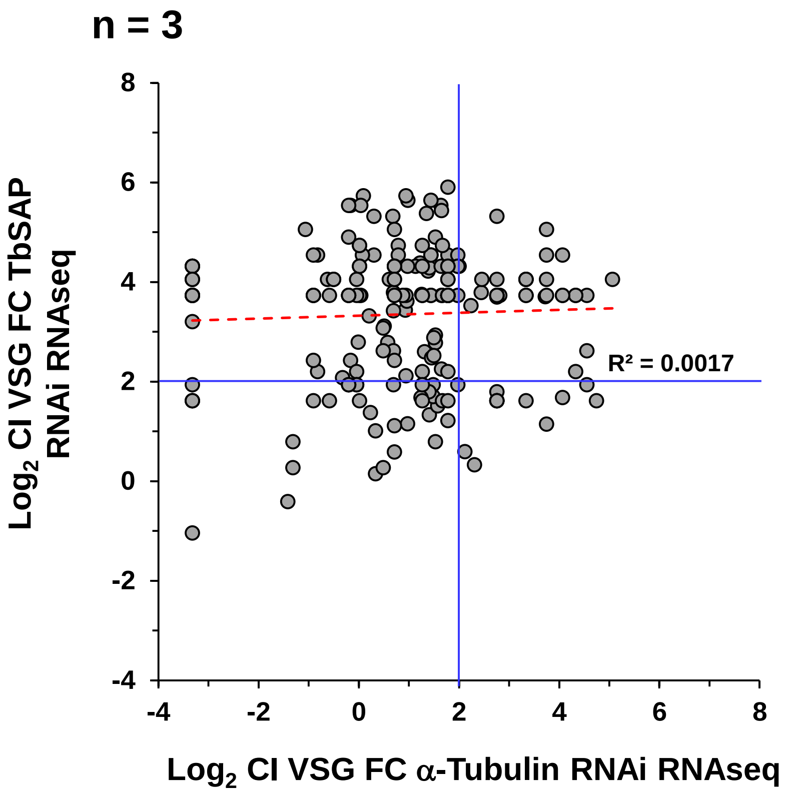

B

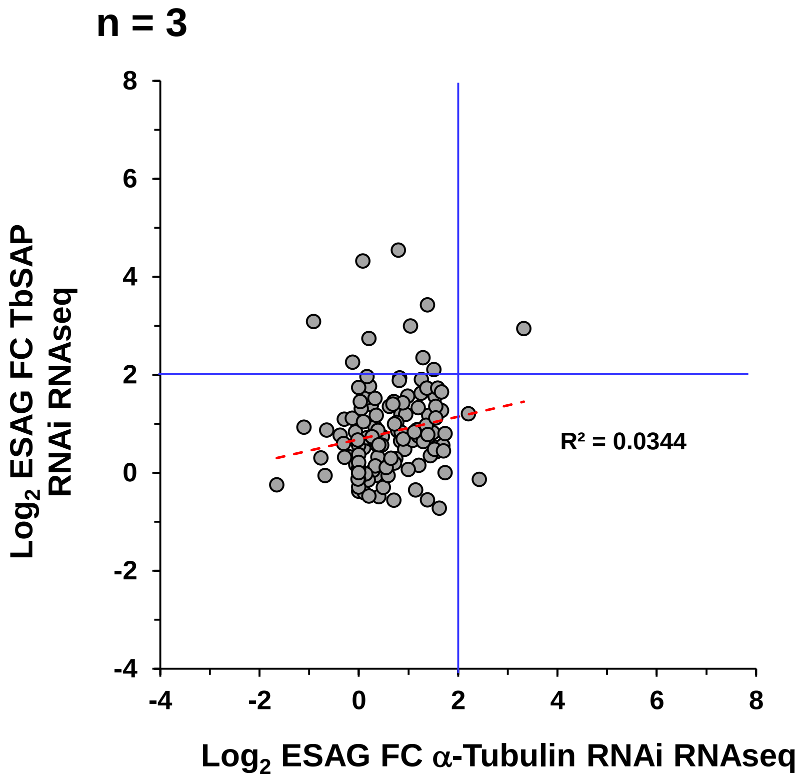

Supplementary Fig. S5

Supplementary Figure S5

The upregulation of silent chromosome internal (CI) *VSG* array genes or *ESAG*s observed after the induction of TbSAP RNAi is not seen after the knockdown of the essential α-tubulin transcript.

(**A**) Comparative analysis of the Log_2 fold change (FC) of chromosome internal *VSG* array gene (CI) transcripts in bloodstream form *T. brucei* after the induction of TbSAP RNAi for 72 hours (Y-axis) compared with transcripts after the induction of α-tubulin RNAi for 16 hours (X-axis). Data are from the means of three biological replicates.

(**B**) As in panel (**A**), only the comparative analysis is of the Log_2 fold change (FC) of *ESAG* transcripts in bloodstream form *T. brucei* after the induction of TbSAP RNAi for 72 hours (Y-axis) compared with the induction of α-tubulin RNAi for 16 hours (X-axis).

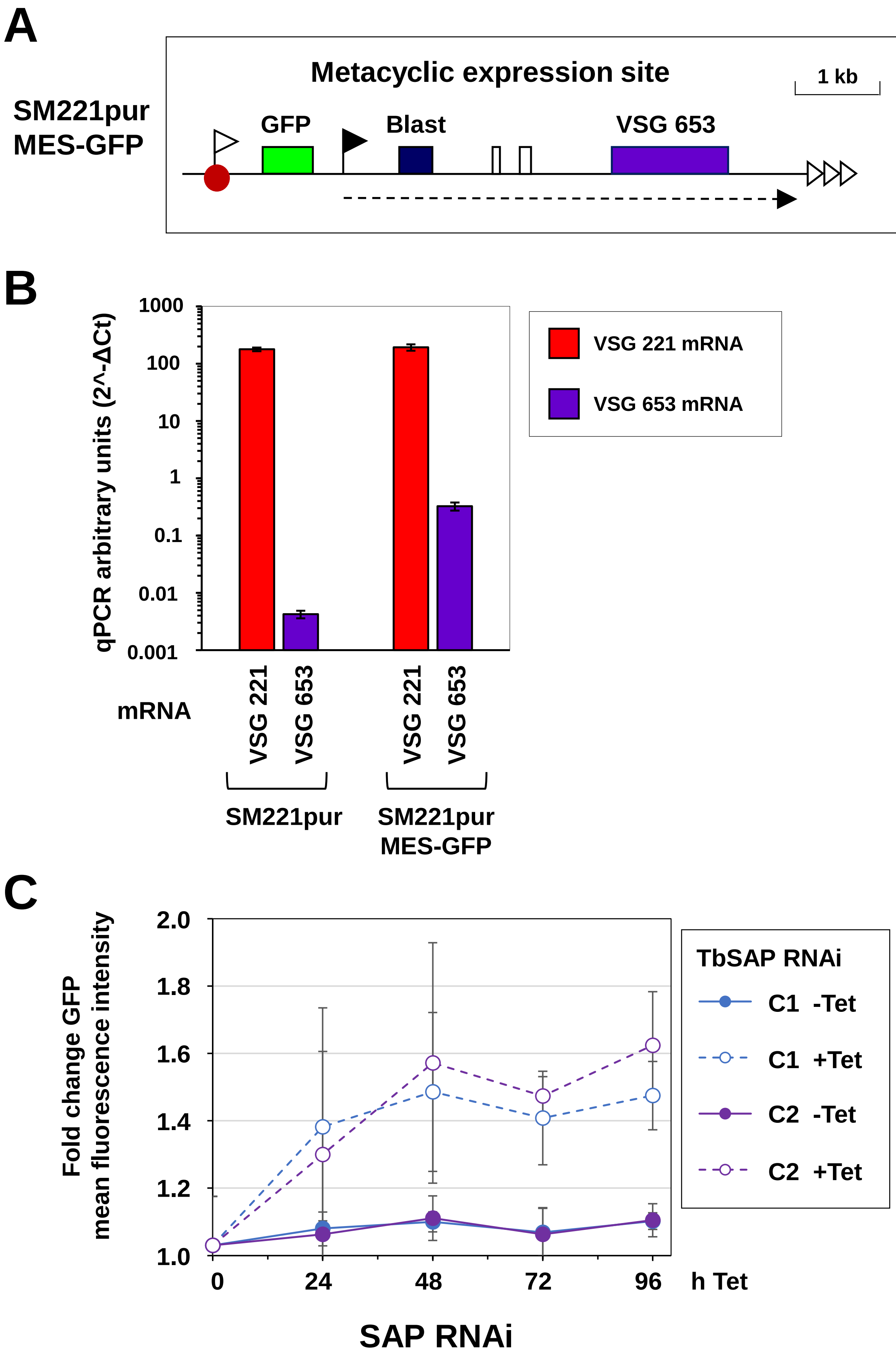
A
Metacyclic expression site
1 kb
SM221pur
MES-GFP
GFP
Blast
VSG 653
B
qPCR arbitrary units (2^-ΔCt)
1000
100
10
1
0.1
0.01
0.001
VSG 221 mRNA
VSG 653 mRNA
mRNA
VSG 221
VSG 653
VSG 221
VSG 653
SM221pur
SM221pur
MES-GFP
C
Fold change GFP
mean fluorescence intensity
2.0
1.8
1.6
1.4
1.2
1.0
0
24
48
72
96
h Tet
SAP RNAi
TbSAP RNAi
C1 -Tet
C1 +Tet
C2 -Tet
C2 +Tet

Sup. Fig. S6

Supplementary Figure S6

Characterisation of the MES-GFP reporter cell line (SM221pur MES-GFP).

(**A**) Schematic of the SM221pur MES-GFP cell line. These cells are bloodstream form *T. brucei* expressing VSG221 from BES1, and have a construct integrated into the metacyclic expression site (MES) *VSG653*. An eGFP gene (green box) is inserted immediately downstream of the endogenous MES promoter (white flag). Integration of the construct was selected for using a blasticidin resistance gene (blast) driven by an ectopic rDNA promoter (black flag). This MES is transcriptionally inactive, with a putative repressor protein on the promoter indicated with a red dot. Transcriptional readthrough from the rDNA promoter (dashed line) results in transcription of the telomeric *VSG653* gene (purple box). Telomere repeats are indicated with horizontal triangles.

(**B**) RT-qPCR analysis of *VSG* transcript from the parental SM221pur or SM221pur MES-GFP cell lines. Transcript from *VSG221* is indicated with red bars, and transcript from *VSG653* with blue bars. Error bars indicate the standard deviation from three biological replicates.

(**C**) Flow cytometry analysis of the mean GFP fluorescence intensity in SM221pur MES-GFP cells in the presence (+) or absence (-) of the induction of TbSAP RNAi with tetracycline (Tet) for the time indicated in hours (h). The fold change in GFP fluorescence intensity from two clones (C1 and C2) is presented, and represent the means of three biological replicates with error bars indicating standard deviation.

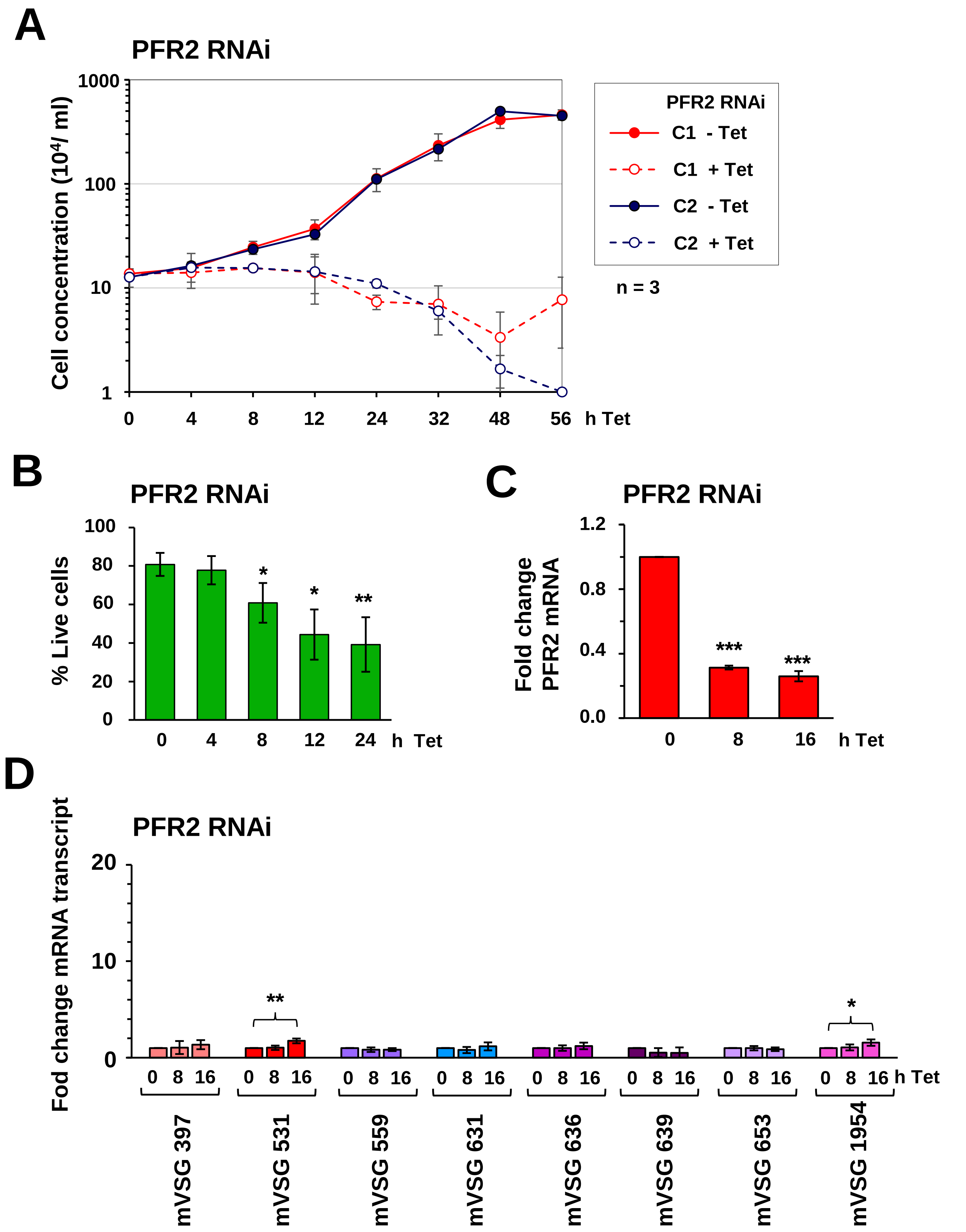
A
PFR2 RNAi
Cell concentration (10⁴/ ml)
1000
100
10
1
0
4
8
12
24
32
48
56
h Tet
PFR2 RNAi
C1 - Tet
C1 + Tet
C2 - Tet
C2 + Tet
n = 3
B
PFR2 RNAi
% Live cells
100
80
60
40
20
0
*
*
**
0
4
8
12
24
h Tet
C
PFR2 RNAi
Fold change
PFR2 mRNA
1.2
0.8
0.4
0.0
***
***
0
8
16
h Tet
D
PFR2 RNAi
Fod change mRNA transcript
20
10
0
**
*
0 8 16
h Tet
mVSG 397
mVSG 531
mVSG 559
mVSG 631
mVSG 636
mVSG 639
mVSG 653
mVSG 1954

Sup. Fig. S7

Supplementary Figure S7

Metacyclic *VSG* expression sites are not significantly derepressed after the induction of RNAi against the essential PFR2 transcript in bloodstream form *T. brucei*.

(**A**) Cell concentration through time after the induction of RNAi against paraflagellar rod protein 2 (PFR2) transcript in ~~of~~ SM221pur MES-GFP PFR2 RNAi cells. Two *T. brucei* clones (C1 and C2) were analysed in the presence (+) or absence (-) of tetracycline (Tet) to induce *PFR2* RNAi for the time indicated in hours (h). Results are the mean of three biological replicates with the standard deviation indicated with error bars.

(**B**) Determination of live/ dead *T. brucei* SM221pur MES-GFP PFR2 RNAi cells after the induction of PFR2 RNAi with tetracycline (Tet) for the time indicated in hours (h). Cells were stained with propidium iodide and analysed by flow cytometry. Data for clone C1 is shown with error bars representing the standard deviation from three biological replicates. Tests for the statistical significance of lethality (t-test) compared time point 0 with different time points after the induction of RNAi, with *P<0.05 and **P<0.01.

(**C**) Quantitation of the degree of PFR2 transcript knockdown in clone 1 (C1) after the induction of PFR2 RNAi. RT-qPCR was performed on RNA isolated from cells where PFR2 RNAi had been induced with tetracycline (Tet) for the time indicated in hours (h). Error bars indicate the standard deviation from the mean from three biological replicates. Statistical significance of the knockdown compared with time 0 using t-tests is shown with significance indicated with ***P<0.001.

(**D**) Induction of a lethal phenotype using PFR2 RNAi leads to little significant upregulation of metacyclic *VSG* genes. RT-qPCR was performed using RNA isolated from cells where PFR2 RNAi had been induced with tetracycline (Tet) for the time indicated in hours (h). Values were normalised against actin and are shown as fold-change relative to the uninduced sample.

Values are plotted as the mean of three independent experiments with the standard deviation indicated with error bars. Statistical significance for upregulation was determined using Student's t-test (*P<0.05, **P<0.01).

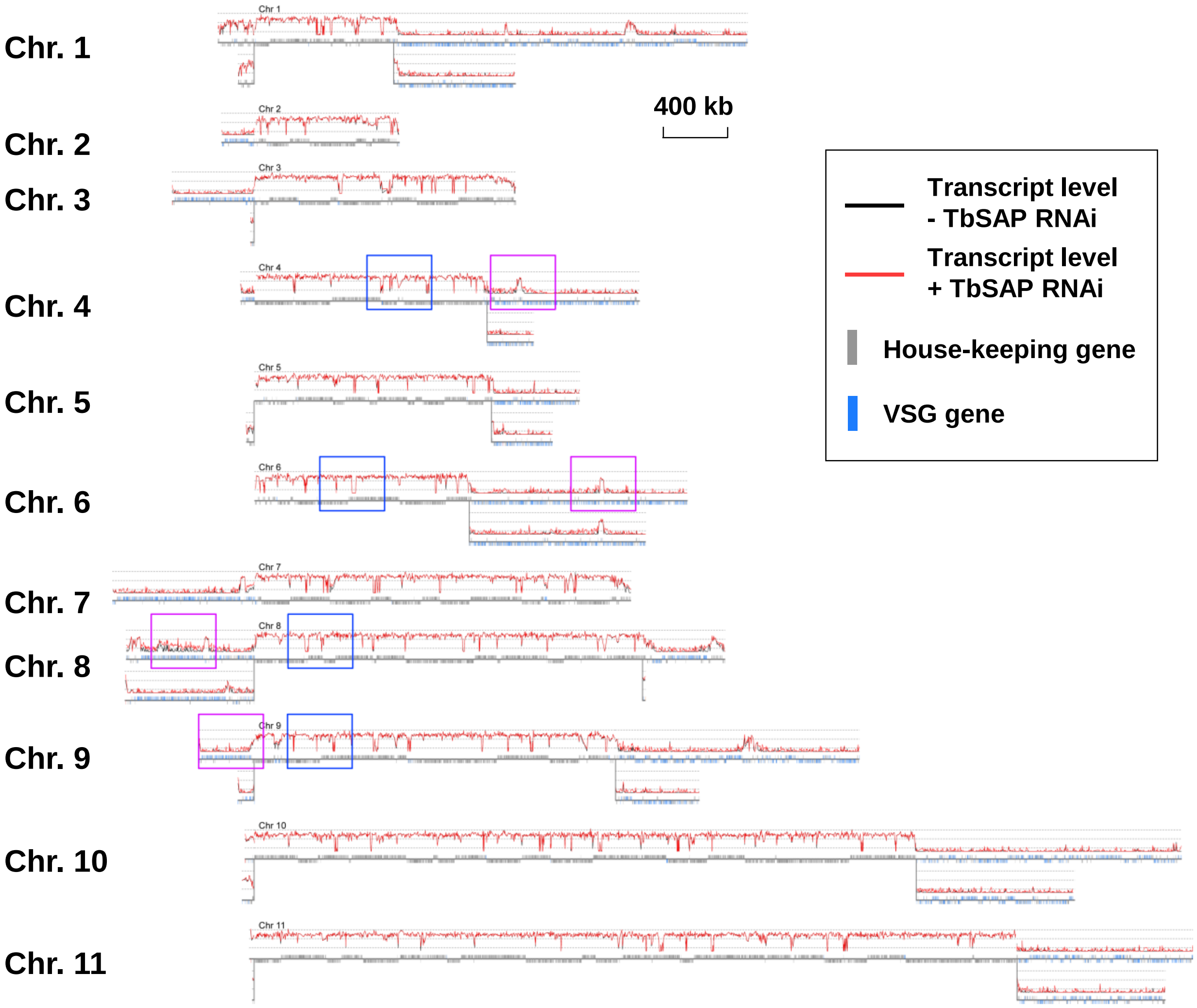

Chr. 1
Chr. 2
Chr. 3
Chr. 4
Chr. 5
Chr. 6
Chr. 7
Chr. 8
Chr. 9
Chr. 10
Chr. 11
400 kb
Transcript level - TbSAP RNAi
Transcript level + TbSAP RNAi
House-keeping gene
VSG gene

Sup. Fig. S8

Supplementary Figure S8

Upregulation of some silent chromosomal internal (CI) *VSG* array genes after the induction of TbSAP RNAi. RNA-seq reads from *T. brucei* SM221pur MES-GFP where TbSAP RNAi had been induced for 72 hours were mapped back to the megabase chromosomes of the *T. brucei* 427 genome (45). Black lines represent transcript reads (mean number from three replicates) from uninduced cells, and red lines transcript reads after induction of TbSAP RNAi. Grey bars represent house-keeping genes and blue bars represent *VSG* genes. Selected magnified regions shown in Figure 9 are indicated with blue boxes highlighting regions of homozygous transcriptionally active chromosomal core regions, and pink boxes highlighting heterozygous transcriptionally silent regions.

Supplementary Table S1. Cell lines and constructs used in this study

Cell line	Integrated construct	Parent	Reference
SM221			Wirtz et. al., 1999
SM221 MES-PurR	pMES653PurRbla	SM221	
SM221pur	pHNES221 Pur1.6	SM221	Stanne *et. al.,* 2011
SM221pur MES-eGFP	pMES653eGFPbla	SM221pur	
SM221pur Cas9	pSMOx2Cas9	SM221pur	
eGFP::TbSAP	PCR product for Cas9-mediated gene tagging. Primers and plasmid template described in Supplementary Table 2.	SM221pur Cas9	
eGFP::TbSAP; Ty1::TRF::Ty	PCR product for Cas9-mediated gene tagging. Primers and plasmid template described in Supplementary Table 2.	eGFP::TbSAP	
eGFP::TbSAP/-	PCR product for Cas9-mediated gene knockout. Primers and plasmid template described in Supplementary Table 2.	eGFP::TbSAP	
SM221pur MES-eGFP TbSAP RNAi	pDexv4xPEX11 TbSAP	SM221pur MES-eGFP	
SM221pur MES-eGFP PFR2 RNAi	pDexv4xPEX11 PFR2	SM221pur MES-eGFP	
BNII VO2+ pSMOx2	pSMOx2	BNII VO2+	BNII VO2+ parental cells previously published in Sheader *et. al.*, 2003
BNII VO2+ pSMOx2 α-Tub RNAi	pDexv4xPEX11 α-Tub	BNII VO2+ pSMOx2	

Supplementary Table S2. Primers and reagents used for Gibson assembly of plasmids (MES integration) and Cas9-mediated tagging/ gene knockout).

**Primers for Gibson assembly of MES targeting plasmid** (Gibson assembly overhangs are in uppercase. Common parent plasmid represents single Gibson assembly step).				
**Parent plasmid**	**Parent plasmid (restriction enzyme digest)**	**Gibson assembly primer**		**Outcome (Resulting plasmid)**
		**Forward**	**Reverse**	
**pMESPurRbla**	**pPFRPurAct (BamHI)**	CGCTCTAGAACTAGTGtaatttttcggctaaacgg	ATAATTGCGGCCACAGtgtcgttctctggtttag	Insertion of MES653 upstream homology region into pPFRPurAct upstream of PFRIR-PURO-ActinIR. (pMESPFRPurAct)
	**pMESPFRPurAct (HindIII)**	TCGTTGCTGCCATAAAATAActttccacccagcgcggg	GACAAGGGAAttagccctcccacacataaccag	Insertion of rDNA promoter-blasticidin resistance gene into pMESPFRPurAct downstream of PFRIR-PURO-ActinIR. (pMESPurRbla)
		GGAGGGCTAAttcccttgtctcgtgtcttttc	TCCAGGATCCagtggcaatgaaacagtaag	Insertion of aldolaseIR into pMESPFRPurAct downstream of rDNA promoter-blasticidin resistance gene. (pMESPurRbla)
		CATTGCCACTggatcctggaggctgtag	CGAGGTCGACGGTATCGATAatatatgcaactctgctcgaac	Insertion of MES653 downstream homology region into MESPFRPurAct downstream of aldolaseIR. (pMESPurRbla)
**pMESeGFPbla**	**peGFPTubbla (NotI, AgeI)**	CTCCACCGCGGTGGCtaatttttcggctaaacgg	CGGCCACAtgtcgttctctggtttag	Excision of βαTubIR from peGFPTubbla and insertion of MES653 upstream homology region. (pUSMESeGFPbla)
		GAACGACAtgtggccgcaattattatg	TCACCATGGTGGCGAaggataatcgatttcttgtgttg	Excision of βαTubIR from peGFPTubbla and insertion of PFRIR downstream of MES653 upstream homology region. (pUSMESeGFPbla)
	**pUSMESeGFPbla (HindIII)**	CGTTGCTGCCATAAAATAAccatcctggaggctgtag	GTCGACGGTATCGATAatatatgcaactctgctcgaac	Insertion of MES653 downstream homology region into pUSMESeGFPbla

				downstream of blasticidin-actinIR. (pMESeGFPbla)
**Primers for Cas9-mediated tagging/gene knockout** (Gene-specific overhangs are in uppercase).				
**Purpose**	**Plasmid template**	**Tagging primers**		**Guide RNA primer**
		**Forward**	**Reverse**	
N-terminal tagging of TbSAP with eGFP	pPOTv6-hyg-hyg-eGFP	ACTGCGGTCGCTTTCGCTCCCATCTGTCCTgtataatgcagacctgctgc	GTACACAATTTTCCGCCCGGGTTTCCTCATactacccgatcctgatccag	gaaattaatacgactcactataggAAGATGAGCTCGGATCAAAGgttttagagctagaaatagc
N-terminal tagging of TbTRF with Ty1	pPOTv6-phleo-blast-10xTY	GGTTGTTCTTTTATATTTTTCTCATATACTgtataatgcagacctgctgc	TGTGGATGGAACGCCAGCGTGACAGTACATactacccgatcctgatccag	gaaattaatacgactcactataggTTGTTGACGTAATTCCAGGGgttttagagctagaaatagc
Single allele knockout of TbSAP	pDexV4-mSt-KIN17 (Phleomycin resistance gene)	ACTGCGGTCGCTTTCGCTCCCATCTGTCCTgggcacagcaaggtcttctg	ACAACGGTGGATACACCCAGTAAGCAAAGAaatactgcatagataacaaa	gaaattaatacgactcactataggAAAGGACAGGAAGAAGGTGAgttttagagctagaaatagc

Supplementary Table S3

**Primers for ChIP quantitation**			
**MES 653 region**			
**Target**	**Forward primer**	**Reverse primer**	**First published**
250 bp upstream of MES	AGCTTTTTCCCACCACGT	CACTATACTCTTTTCCGCTTGG	
MES promoter core	CCTATAACCCGACCAAATA	GACGGGTTAGTCTTCTTTCT	
mVSG 397	CTGGAACAACAGGCACAAGC	CAGTCCTGTCAAGATGCCGT	
mVSG 653	GTACCTACGCCGAGCTTCAA	CACTGAATTTGCCCGTCGTG	
**BES region**			
**Target**	**Forward primer**	**Reverse primer**	**First published**
~600 bp upstream of BES promoters	TATTATGGATCAGGTCAGAG	AACATACTGCAACAACAATC	Stanne and Rudenko, 2010
~200 bp upstream of BES promoters	GGTTAGAATCACTATAATGC	AGAACGCCTCATAACTCT	
~150 bp upstream of BES promoters	GGATAATACATTTGTCGCAGCT	AGTTAACAATTAAGACAGCG	
BES promoter core	TATCCGAGATTTCTGCAA	ATATCCCTATTACCACACCA	Stanne and Rudenko, 2010
Puromycin resistance gene	CGAGTTGAGCGGTTCC	GCCTTCCATCTGTTGCT	Denninger and Rudenko, 2014

ESAG 6/7	AACAGTATTGAGGAATGAG	ATTTTGTAAAGGGTTTCAG	Stanne and Rudenko, 2010
Tb427VSG-2 (VSG 221)	GCGACAACCAGCCAACCAAG	TCAGCGGGCTTGTGCTTCTG	Ridewood *et. al.*, 2017
Tb427VSG-18 (VSG 800)	GAAGGTCTGGGAACCTCTAT	GGCTGTAATATGCTCGTAGA	Budzak *et. al.*, 2019
**rDNA region**			
**Target**	**Forward primer**	**Reverse primer**	**First published**
600 bp upstream of rDNA promoter	CCGGAGTAGCCTTTCCAGCG	GAGCAGGCGCCAATGTCAAC	
rDNA promoter core	GTACGGAGCAGGAGAGCAAC	GCATTGCGCAAAGTTTACAG	Stanne and Rudenko, 2010
18S rDNA	GCATTACTGGATAACTTGG	GTTCTAATTTCATTCATTCG	Stanne and Rudenko, 2010
28Sα rDNA	ACACATTTACAACCCTTCAT	CTATCGGTCTTCCTACTCTAT	Stanne and Rudenko, 2010
rDNA spacer	ATTTTCTCTACCCCTCTCTT	ATCATCGTATCATTTTCATC	Stanne and Rudenko, 2010
**Actin**			
	**Forward primer**	**Reverse primer**	
	GTTCCATCCTCTCATCACTA	TCGTATTCACTCTTCGTTATC	

Supplementary Table S4

**Primers for qPCR quantitation of mRNA**		
**Target**	**Forward primer**	**Reverse primer**
Actin	GTTCCATCCTCTCATCACTA	TCGTATTCACTCTTCGTTATC
TbSAP	CTCAAGACTGTTCCGAGCGT	GCGCTTGAGTCCCGATACAT
PFR2	CCGACAAGAAGAAAG ACCTATACAA	GTTCAGCGCATCCTCAGTAG
VSG221	GCGACAACCAGCCAACCAAG	TCAGCGGGCTTGTGCTTCTG
mVSG 397	CTGGAACAACAGGCACAAGC	CAGTCCTGTCAAGATGCCGT
mVSG 531	TTGGGGGAACTCCTGAGCTA	GCTTTACCCCCTGCTCCTTT
mVSG 559	GGTCTGAAGCAAACAGCGTG	CTTTTAAGTACAGCGCCGCC
mVSG 631	CCCTGACATACCATGGGTCG	TTCTAGAGCTTGTGCGTCGG
mVSG 636	CGGCACTGGTGACAACAAAT	TTAAAAGGCCCAGCAACGTG
mVSG 639	GGCTCAGCGAGCAGAACTAT	GCAGAGCTGAAAGTGCGAAG
mVSG 653	GTACCTACGCCGAGCTTCAA	CACTGAATTTGCCCGTCGTG
mVSG 1954	GCCTAGCGATAAGAGGGGAC	TTCTTGGCAATGTCGCTGTC

Supplementary Table S5.

MES VSG mRNA fold change 72 h post-TbSAP RNAi quantitated by RNAseq or qPCR.

Data for RNAseq and qPCR analysis are from 3 biological replicates.

**mVSG**	**Genome ID**	**RNAseq data**		**qPCR data**
		**RNAseq FC**	**Adj. P value**	**qPCR FC (± SD)**
mVSG 397	Tb427_000106600	10.6 X	0.025	16.2 ± 3.0 X
mVSG 531	Tb427_000304300	6.5 X	0.033	8.0 ± 1.5 X
mVSG 559	Tb427_000615900	12.3 X	8.3×10^{-13}	9.3 ± 3.8 X
mVSG 582	Tb427_000369100	10	2.4×10^{-9}	
mVSG 631*	Tb427_000108300, Tb427_000432500	0.9 X	1	6.0 ± 0.7 X
mVSG 636	Tb427_000627000	18.5 X	2.6×10^{-3}	25.6 ± 7.7 X
mVSG 639	Tb427_000288600	14.0 X	3.5×10^{-4}	11.0 ± 4.0 X
mVSG 653**	Tb427_000173600	2.0 X	0.086	2.1 ± 0.1 X
mVSG 1954	Tb427_000524500	17.6 X	1.4×10^{-3}	16.8 ± 3.2 X

*VSG-631 is present as two copies in the genome, of which only Tb427_000108300 is situated downstream of a possible MES promoter (8.9 kb from start of ORF).

**VSG 653 is present within the marked MES in the reporter strains.

nd= not determined

Supplementary File S1

High confidence hits identified by RIT-seq analysis following puromycin selection of the MES RNAi library. Reads derived from Illumina sequencing of PCR amplified RNAi target fragments were mapped to the *T. brucei* strain 927 reference genome and converted to reads per kilobase per million reads mapped (RPKM) to enable comparison between the three RNAi library screens. Only the hits corresponding to an RPKM equivalent of at least 100 reads (139 or 142, respectively) following selection in 50 or 60 ng/ml puromycin are presented. RPKM data for the corresponding hits following selection in 70 ng/ml puromycin are shown where these values match or exceed the 100 read equivalent RPMK cut-off for this screen (212). Fitness and procyclic localisation data for each hit is derived from [PMID21363968] and Tryptag.org [PMID27863903], respectively.

Supplementary File S2

Processed RNA-Seq data. Data have been mapped to the Lister 427 long-read assembly (427_2018; TriTrypDB v46). See Materials and Methods for details of processing, mapping and analysis. Raw read data are available in the NCBI GEO repository under project number GSE160713. Data show total mapped fragment counts for biological triplicates of experimental conditions, in addition to mean $\log_2$ abundance, $\log_2$ fold-change, Benjamini-Hochberg adjusted p-value and directional $\log_{10}$ q-value. Highlighting in column Q shows genes considered to be differentially expressed at a threshold of adjusted p-value ≤ 0.1 and fold-change ≥ 1.4 (DE = 1 and -1 for up- and down-regulation, respectively).
